# An *in vivo* and *in silico* evaluation of the hepatoprotective potential of *Gynura procumbens*: A promising agent for combating hepatotoxicity

**DOI:** 10.1371/journal.pone.0291125

**Published:** 2023-09-15

**Authors:** Tanzia Islam Tithi, Md. Rafat Tahsin, Juhaer Anjum, Tasnuva Sharmin Zaman, Fahima Aktar, Nasiba Binte Bahar, Sabiha Tasnim, Arifa Sultana, Ishrat Jahan, Syeda Sadia Afrin, Tahmina Akter, Priyanka Sen, Fahima Jannat Koly, Md. Selim Reza, Jakir Ahmed Chowdhury, Shaila Kabir, Abu Asad Chowdhury, Md. Shah Amran

**Affiliations:** 1 Department of Pharmaceutical Technology, Faculty of Pharmacy, University of Dhaka, Dhaka, Bangladesh; 2 Department of Pharmaceutical Sciences, North South University, Dhaka, Bangladesh; 3 Molecular Pharmacology and Herbal Drug Research Laboratory, Department of Pharmaceutical Chemistry, Faculty of Pharmacy, University of Dhaka, Dhaka, Bangladesh; 4 Department of Pharmacy, University of Asia Pacific, Farmgate, Dhaka, Bangladesh; 5 Department of Pathology, Dhaka Medical College, Dhaka, Bangladesh; 6 Department of Physiology, Dhaka Medical College, Dhaka, Bangladesh; 7 Department of Clinical Pharmacy and Pharmacology, Faculty of Pharmacy, University of Dhaka, Dhaka, Bangladesh; 8 Department of Pharmacy, Faculty of Pharmacy, University of Dhaka, Dhaka, Bangladesh; Helwan University, EGYPT

## Abstract

**Introduction:**

The liver, the most important metabolic organ of the body, performs a wide variety of vital functions. Hepatic cell injury occurs by the activation of reactive oxygen species (ROS) that are generated by carbon tetrachloride (CCl_4_), xenobiotics, and other toxic substances through cytochrome P450-dependent steps resulting from the covalent bond formation with lipoproteins and nucleic acids. Observing the urgent state of hepatotoxic patients worldwide, different medicinal plants and their properties can be explored to combat such free radical damage to the liver. *In vivo* and *in silico* studies were designed and conducted to evaluate the antioxidant and hepatoprotective properties of *Gynura procumbens* in rats.

**Materials and methods:**

*Gynura procumbens* leaves were collected and extracted using 70% ethanol. The required chemicals CCl_4_, standard drug (silymarin), and blood serum analysis kits were stocked. The *in vivo* tests were performed in 140 healthy Wister albino rats of either sex under well-controlled parameters divided into 14 groups, strictly maintaining Institutional Animal Ethics Committee (IEAC) protocols. For the histopathology study, 10% buffered neutral formalin was used for organ preservation. Later the specimens were studied under a fluorescence microscope. *In silico* molecular docking and absorption, distribution, metabolism, excretion, and toxicity (ADMET) studies were performed, and the results were analyzed statistically.

**Results and discussion:**

Gynura procumbens partially negate the deleterious effect of carbon tetrachloride on normal weight gain in rats. The elevated level of serum glutamate pyruvate transaminase (SGPT), serum glutamate oxaloacetate transaminase (SGOT), alkaline phosphatase (ALP), creatinine, LDH, total cholesterol (TC), low-density lipoprotein (LDL), triglycerides (TG), malondialdehyde (MDA), deoxyribonucleic acid (DNA) fragmentation ranges, gamma-glutamyl transferase (γ-GT) in CCl_4_ treated groups were decreased by both standard drug silymarin and *G*. *procumbens* leaf extract. We have found significant & highly significant changes statistically for different doses, here p<0.05 & p<0.01, respectively. On the other hand, *G*. *procumbens* and silymarin displayed Statistically significant (p<0.05) and high significant(p<0.01) increased levels of HDL, CAT SOD (here p<0.05 & p<0.01 for different doses) when the treatment groups were compared with the disease control group. Because the therapeutic activity imparted by plants and drugs accelerates the movement of the disturbed pathophysiological state toward the healthy state. In the molecular docking analysis, *G*. *procumbens* phytoconstituents performed poorly against transforming growth factor-beta 1 (TGF-β1) compared to the control drug silymarin. In contrast, 26 phytoconstituents scored better than the control bezafibrate against peroxisome proliferator-activated receptor alpha (PPAR-α). The top scoring compounds for both macromolecules were observed to form stable complexes in the molecular dynamics simulations. Flavonoids and phenolic compounds performed better than other constituents in providing hepatoprotective activity. It can, thus, be inferred that the extract of *G*. *procumbens* showed good hepatoprotective properties in rats.

## Introduction

The liver is a vital and resilient organ in an animal’s body, the primary functions of which are to support metabolism, digestion, detoxification, storing vitamins, minerals, and immunity. However, this organ can be affected by toxins from food supplements, drugs, chemicals, and medicinal plants [1–3]. Carbon tetrachloride (CCl_4_) is considered quite toxic and xenobiotic to the animal body and causes cell injury by activating reactive oxygen species (ROS). Peroxy trichloromethyl (OOCCl_3_) and trichloromethyl (CCl_3_) radicals are usually generated during the cytochrome P450-dependent metabolic steps. These free radicals produce covalent bonds with lipoproteins and nucleic acids that cause extensive cell injury in the liver and other vital animal body organs [4–9]. To date, around fifty million people worldwide are suffering from hepatotoxicity. This hepatotoxicity state is quite alarming in the health sector [1, 10]. Symptoms related to hepatotoxicity may include jaundice, fatigue, abdominal pain, skin rashes, itching, rapid and abnormal weight gain, vomiting or nausea, light-colored stool, and dark urine [11, 12]. To examine liver function for diagnostic purposes, serum glutamate pyruvate transaminase (SGPT), serum glutamate oxaloacetate transaminase (SGOT), and alkaline phosphatase (ALP) levels are measured in the blood [13]. Synthetic drugs such as thalidomide, curcumin, ademetionine, entecavir, metadoxine, tenofovir, ondansetron, and resveratrol are available in the pharma arena, which are used to treat liver diseases [14]. However, the toxicity and efficacy of these medicines are still under investigation, and medicinal plants have an excellent opportunity to take their position through genetic modification using different biotechnological studies [15]. Medicinal plants have always significantly impacted medicines and nutrition for different societies in the world. Around 70,000 plant species have been used for diseases as medicine. Their usage rate in the conventional medicinal system is 25% [16, 17]. In developing countries like China, India, Pakistan, and Sri Lanka (more than 80%) and in developed countries such as the United States (more than 25%), drugs are constituted from medicinal plants. These medicinal plants are a goldmine of potentially therapeutic novel natural substances that can be a poison for combating several fatal diseases. This issue has urged the researchers to pursue natural resources to treat hepatic ailments. In this pursuit, researchers have discovered a plethora of medicinal plants with impressive hepatoprotective actions, including *Annona squamosa*, *Cassia roxburghii*, *Cajanus cajan*, *Coccinia grandis*, *Ficus carica*, *Glycosmis pentaphylla*, *Gynura procumbens*, *Solanum nigrum*, *Sargassum polycystum*, *Tinospora crispa*, *Piper chaba* etc. [18–28]. Among these plants, *Gynura procumbens* is an extraordinary herb widely available in the southeast region of the world, such as Thailand, Malaysia, China, Vietnam, Bangladesh, and Indonesia [29]. It is a small (around 1–3 m long) flowering plant that belongs to the Asteraceae family. This herb is taken as a vegetable item in meals and used as a medicinal plant in these regions. The principal pharmacological activities of *G*. *procumbens* are diabetes, Herpes Simplex Virus (HSV), hyperlipidemia, bacterial infection, inflammation, cancer, analgesic, kidney disease, rheumatism, and hypertension [30–37]. In addition, different bioactive molecules such as polysaccharides, saponins, sterols, tannins, terpenoids, flavonoids, and glycosides are functional chemical components to successfully treat these sorts of diseases [37–42].

Moreover, the most recent revelation of the antioxidant properties of this plant gives a clear and precise idea to treat various diseases by reducing free radical production [33, 43–47]. For this reason, the Chinese name of this plant is "Pokok Sambung Nyawa," the meaning of which is "prolongation of life" [37, 48]. Furthermore, certain findings have provided significant evidence demonstrating the promising hepatoprotective potentials of G. *procumbens*, which has piqued the interest of researchers to investigate further the protective effects of *G*. *procumbens* against specific types of hepatotoxicity [24, 49]. Hence, the present study aims to evaluate the hepatoprotective activities of *G*. *procumbens* against CCl_4_-induced hepatotoxicity, and histopathological studies, and computationally predict the ADMET properties of its phytoconstituents.

## Method and materials

### Collection and extraction of *Gynura procumbens* leaf

*Gynura procumbens* leaves were collected from the medicinal plant garden of the Faculty of Pharmacy, University of Dhaka. After that, the Bangladesh National Herbarium certified the specimen and provided the accession number YM001 for future reference.

The collected leaves of Gynura *procumbens* were then carefully washed and kept for 7 days for shed drying. Next, the dried leaf was kept in an oven at 30°C temperature for 10 days. After that, the dried leaf was powdered coarsely. After that, powdered leaves were extracted using 70% ethanol for twelve days, and the extract was filtered every three days. Later, a rotary evaporator was used to dry the extract using low temperature and pressure. Finally, different pharmacological tests were performed using this crude residue.

### Drugs and chemicals

Carbon tetrachloride (CCl_4_) was bought from the Sigma Aldrich Company, USA. A standard hepatoprotective drug named silymarin (Brand name: silybin) was obtained as a gift from Square Pharmaceuticals Ltd. Again, SGPT, SGOT, ALP, Creatinine, Total Cholesterol, HDL, LDL, Triglyceride were assessed by using blood serum analyzing kits, which were bought from Orbit Trade Ltd, and Gentech Ltd, Bangladesh. These parameters were analyzed by Humalyzer 3000, Germany [50, 51]. LDH, CAT, SOD, and MDA were measured by maintaining the parameters provided by Jianchueng Biochemical Technological Institute, Nanjing [52]. γ-GT were calculated using standard AMP diagnostic kits from Stattogger Strasse 31b 8045 Graz, Austria [53]. Later, different groups’ oxidative stress parameters and DNA fragmentation were completed by using Diphenylamine reaction procedure, and the calculation was conducted with the help of the following formula:

%FragmentedDNA=[OD(S)∕[OD(S)+OD(P)]×100

(OD: optical density, S: supernatants, P: pellets) [54].

### Sample size determination

For every animal model research, the “Power Analysis Method” is used to determine the sample size. The following formula can be used to perform the computation manually.

Samplesize=2SD2(Zα/2+Zβ)2/d2

Where standard deviation is obtained from previous studies or initial pilot studies.

Now,

$$\begin{array}{cc} \text{Z}\alpha /2 & =\text{Z}\times 0.05/2\\ & =\text{Z}\times 0.025\\ & =1.96\;\text{at}\;\text{type}\;1\;\text{error}\;\text{of}\;5\%\;\left(\text{From}\;\text{Z}\;\text{table}\right)\\ \text{Z}\beta & =\text{Z}\times 0.20=0.842\;\text{at}\;80\%\;\text{power}\;\left(\text{From}\;\text{Z}\;\text{table}\right) \end{array}$$

Here, d = difference between mean values.

The final sample size was modified to account for anticipated attrition. According to our previous study, it was seen that 10% of rats may die as a result of disease-inducing processes. So, in order to get the actual sample size, we divided the total by 0.9.

The pilot study employed Five rats as the disease control group (CCl4-induced group). A single oral dose of CCl4 in combination with olive oil as the vehicle in a 1:1 ratio (3 ml/kg of rat body weight) was administered to induce hepatotoxicity. After a specific study period, SGPT levels were determined to be 70.16 U/L on average, with a standard deviation of 4.95.

According to our previous research, it can be considered that if the mean SGPT level is found to be 63.61 U/L following treatment with the extract, then it can be concluded that the plant extract may considerably lower the increased SGPT level (p<0.05).

So, the standard deviation of the pilot study was 4.95.

And,

$$\begin{array}{cc} \text{Z}\alpha /2 & =1.96,\\ \text{Z}\beta & =\text{Z}\times 0.20=0.842,\\ \text{d} & =70.16-63.61=6.45. \end{array}$$

So, sample size = 2 SD2 (Zα/2 + Zβ)2/d2 = 2× (4.95)2× (1.96 + 0.842)2/(70.16–63.61)2 = 8.97.

The ultimate sample size must now be modified to account for anticipated attrition. Thus, our sample size will be 8.97/0.9 = 9.97. As the sample size cannot be a fraction, ten rats were taken in each group [51].

### Health status of animal model

Knowing the health condition of rodents is crucial for ensuring valid and reproducible research results. To ensure safety and efficacy, we have monitored the health status of all our rodents and compiled them in a scoresheet for scoring endpoints. We have checked the appearance, loss of weight, behavior, and locomotion of rats. Maximum rodents had shown the normal score, a very few of them scored differently than normal ones. All the parameters and scoring were done according to the code of practice for the housing and care of laboratory animals [55, 56].

### Animals

One hundred forty (140) healthy male rats (Wistar rats) weighing between 120–180 g were purchased from Jahangirnagar University, Dhaka. Each of them was nourished carefully by keeping them in the Institute of Nutrition & Food Science at the University of Dhaka in a well-controlled environment (relative humidity 55±5%, 12±1h light/dark cycle, and temperature 25±3°C) for two weeks. A total of 10 rats were selected for each group, which included 3 rats from the range 121-140gm, 4 rats from 141–160 g, and 3 rats of 161-180g body weight. All rats were provided with a standard food supplement and purified water. Finally, all experimental procedures were carried out according to the Institutional Animals Ethics Committee (IEAC) protocols.

### CCl_4_ preparation for gastric lavage

A single oral dose of CCl_4_ in combination with olive oil as the vehicle in a 1:1 ratio (3 ml/kg of rat body weight) was administered in respective groups regularly. Both drug and plant extract were administrated orally. Gynura *procumbens* extract (500, 750 & 1000 mg/kg) and Silymarin (80, 120, 150 mg/kg) were induced to ease the hepatic damage. CCl_4_, Silymarin, and plant extract animals were administered using a gastric tube. Animal grouping and treatment procedures are shown in Table 1.

**Table 1:** Protocol for evaluation of the hepatoprotective activity.

Group Number	Group Status	Treatment Specimen	The volume of Treatment specimen (mg/kg)	Group Abbreviation
**1**	Negative Control	Physiological Saline	10mL/kg	C
**2**	Disease control	Carbon tetra chloride (CCl_4_)	3mL/kg	CCl_4_
**3**	CCl_4_ + Silymarin	Silymarin	3mL/kg+80mg/kg	CCl_4_+S_80_
**4**	CCl_4_ + Silymarin	Silymarin	3mL/kg+120mg/kg	CCl_4_+S_120_
**5**	CCl_4_ + Silymarin	Silymarin	3mL/kg+150mg/kg	CCl_4_+S_150_
**6**	CCl_4_ + *Gynura procumbens*	*Gynura Procumbens*	3mL/kg+500mg/kg	CCl_4_+GP_500_
**7**	CCl_4_ + *Gynura procumbens*	*Gynura Procumbens*	3mL/kg+750mg/kg	CCl_4_+GP_750_
**8**	CCl_4_ + *Gynura procumbens*	*Gynura Procumbens*	3mL/kg+1000mg/kg	CCl_4_+GP_1000_
**9**	Silymarin	Silymarin	80mg/kg	S_80_
**10**	Silymarin	Silymarin	120mg/kg	S_120_
**11**	Silymarin	Silymarin	150mg/kg	S_150_
**12**	*Gynura procumbens*	*Gynura Procumbens*	500mg/kg	GP_500_
**13**	*Gynura procumbens*	*Gynura Procumbens*	750mg/kg	GP_750_
**14**	*Gynura procumbens*	*Gynura Procumbens*	1000mg/kg	GP_1000_

Group 2–8 was administered with carbon tetrachloride (CCl_4_) at a 3 mL/kg dose through an intraperitoneal route to induce hepatotoxicity. After the treatment, all rats were sacrificed, and blood was collected by cardiac puncture, Then different parameters such as Serum Glutamate Oxaloacetic Transaminase (SGOT) and Serum Glutamate Pyruvate Transaminase (SGPT), Alkaline phosphatase (ALP), Gamma-glutamyl transferase (γ-GT), Creatinine, Total Cholesterol (TC), High-density lipoprotein (HDL), Low density lipoprotein (LDL), Triglyceride (TG), Catalase (CAT), Superoxide dismutase (SOD) and Malondialdehyde (MDA) were measured in all groups to check the effect of CCl_4_, Silymarin and plant extract on pathological condition of rat. The duration of treatment was six weeks.

### Histopathological studies

The liver tissues of the experimental rats were carefully excised and rinsed with a normal saline solution. The liver samples were then preserved in 10% buffered neutral formalin for about 48 hours. After proper fixation, grossing is done. A gross section has been taken from the representative area. Then it is again fixed in formalin, then a paraffin-embedded block is prepared. After sectioning and staining with hematoxylin and eosin, a very thin, high-quality section was mounted on glass slides. After that, it is observed under a microscope at low and high power view, and the findings are identified.

### *In silico* molecular docking, molecular dynamics, and ADMET studies

A list of seventy-six (76) phytoconstituents of *G*. *procumbens* was prepared by thoroughly inspecting previous literature. These phytoconstituents comprised the ligand library for the *in-silico* assessment of hepatoprotective activity. 3D coordinate files of the constituents were either downloaded from the ’PubChem’ or the ’ChemSpider’ databases or drawn manually on the ‘Avogadro’ software package [37, 48]. The macromolecules TGF-β type I Receptor (PDB ID: 1VJY) and PPAR-α (PDB ID: 5HYK) were selected as the molecular targets for the docking studies, as these have been reported to be potent molecular mediators and regulators of hepatoprotective action [50, 51, 57, 58]. The active sites of the macromolecules were deduced from literature, and galunisertib and bezafibrate were employed as control molecules, respectively [59–62]. The phytoconstituents and the control molecules were optimized under the MMFF94 force field employing the steepest descent algorithm (convergence value: 10e^-7^) using the software package ’Avogadro’ and saved in the PDB format [63]. The macromolecules were downloaded from the ’Protein Data Bank’ database and prepared using the software packages ’PyMol’ and ‘Swiss-PdbViewer 4.1.0’, the latter of which was utilized to carry out energy minimization using the ‘GROMOS96’ force field with parameters set 43B1 in vacuo [64–67].

The Autodock Vina component of the software package ‘PyRx’ was used to carry out molecular docking [68]. Results were visualized and analyzed in the software packages PyMol’ and ‘Discovery Studio Visualizer 2020’ [64, 69].

To assess the stability of the protein ligand complexes obtained from the molecular docking study, 100 nanosecond molecular dynamics simulations were conducted on the top performing ligands of each macromolecular target, along with their non-ligand bound form. The Desmond software suite was utilized for this, along with the OPLS3e forcefield [70, 71]. To conduct the molecular dynamics simulation, 10 Å×10 Å×10 Å orthorhombic simulation boxes were generated, and isotonic saline solutions were emulated along with additional Na^+^ or Cl^-^ ions the neutralize the net charge of the system. NPT ensemble was utilized at 300 K temperature and 1.02325 base pressure, and snapshots were taken every 100 picoseconds to obtain 1000 frame trajectories [72].

The computational web server ‘SwissADME’ was used to predict the ADME properties of the phytoconstituents, and the webserver ‘ProTox-II’ was utilized to predict the toxicity status of the same [73–77].

### Statistical analysis

All our findings (raw data) belong to several groups regarding numerous research parameters recorded and analyzed on a broadsheet using an MS Excel program. Data were subjected to descriptive statistics, and results were represented as mean±SD. We employed the "One Way Anova Test’’ of "SPSS 16" software to interpret the inter-group heterogeneity in diverse biological parameters to determine the statistical significance. We consider the events as statistically significant and highly significant, while the p-value was detected as less than 0.05 (p<0.05) and 0.01 (p<0.01), respectively.

### Ethical statement

This study has been approved (Ref. no. 146/Biol. Scs.) by the Ethical Review Committee of the Faculty of Biological Sciences, University of Dhaka.

## Results

**Fig 1:**
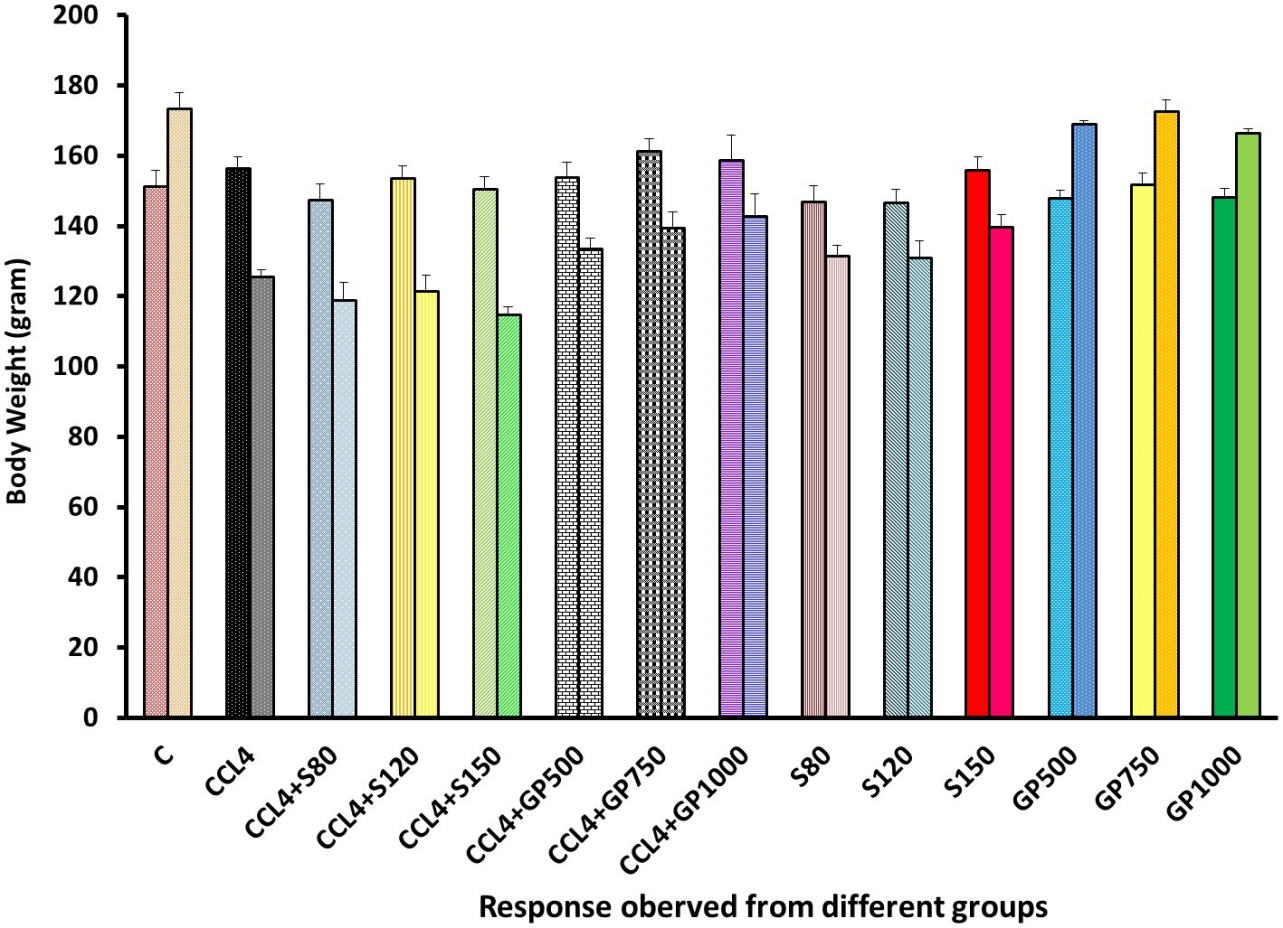
Effect of various treatment specimens on the body weight of CCl4- treated rat. Comparison between the average body weight (mean ± standard deviation) of rats belonging to 14 groups before starting the experiment and just before sacrifice. Each group consists of 10 rodents each with equal body mass index. X axis represents the group distribution and y-axis represents the body weight in grams of different groups. All abbreviation of different groups has been mentioned in Table 1.

A significant elevation of body weight was observed after administering *Gynura procumbens* extract in CCl_4_-induced hepatotoxic rats [Fig 1]. In the negative control group, body weight was increased at the final stage. However, carbon tetrachloride treatment prompted a decrease in terminal body weight in the disease control group. Groups treated with three different doses of *Gynura procumbens* (low, medium, and high) following carbon tetrachloride elevated the gradual pattern of weight gain. However, silymarin treatment showed the opposite scenario. A reversal of CCl4-mediated body weight decline was reported upon treatment with different doses of *Gynura procumbens* extract. Groups administered only plant extract followed a similar trend as the negative control group, whereas a contrasting scenario was observed in groups given silymarin only.

**Fig 2:**
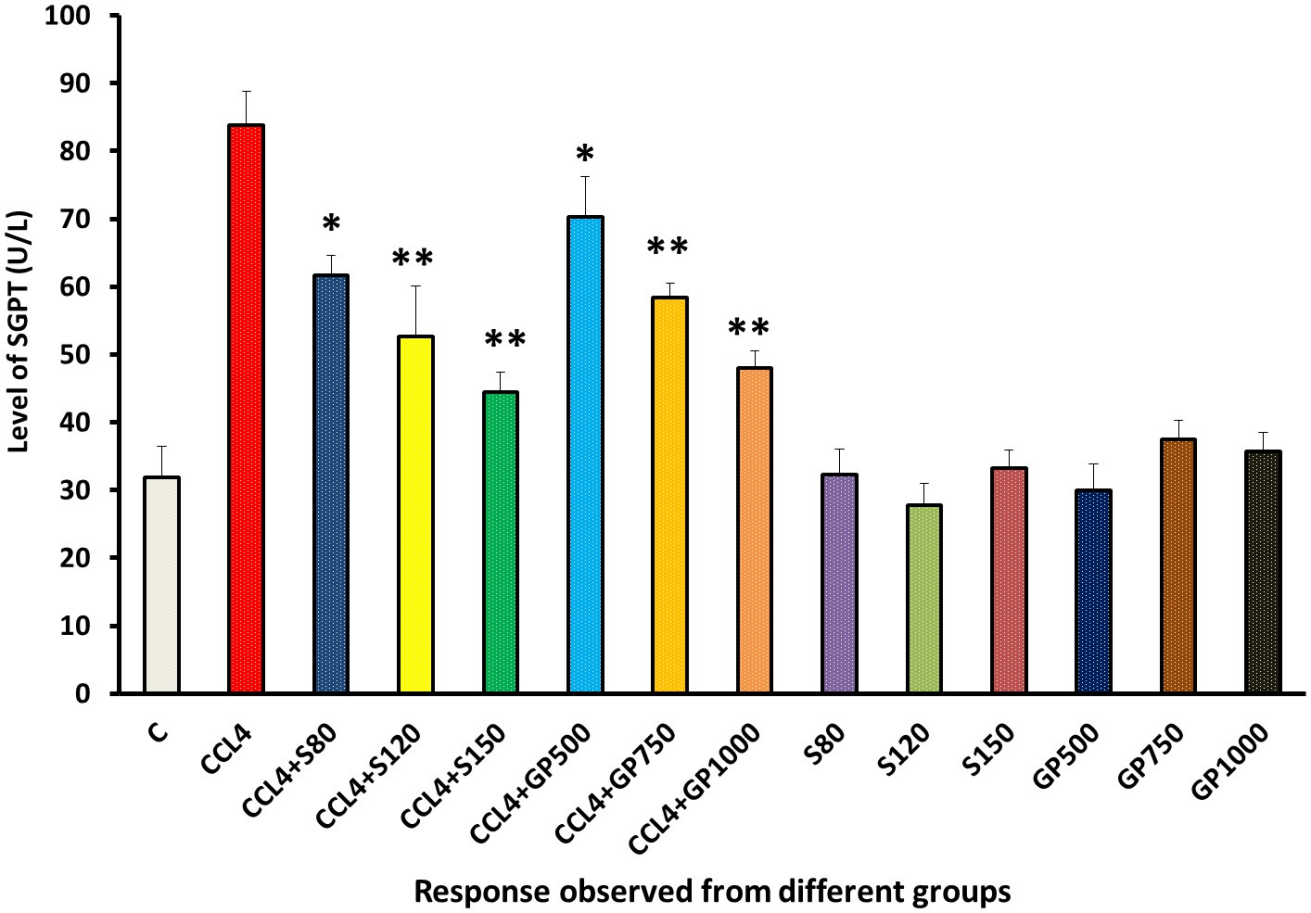
Effect of various treatment specimens on SGPT level of CCl4- treated rats. Comparison of SGPT level (U/L) of rats, belonged to 14 groups just before sacrifice. Each group consist of 10 rodents each with equal body mass index. The data were expressed as mean±standard deviation. X axis represents the group distribution and y-axis represents SGPT level of different groups. All abbreviation of different groups has been mentioned in Table 1. (*indicates statistically significant change where p<0.05, correlation is significant at a 95% confidence interval and **indicates highly significant change where p<0.01, correlation is significant at a 99% confidence interval).

The SGPT level of the carbon tetrachloride-treated group was higher than that of the negative control. SGPT levels were significantly (p<0.05) reduced by drugs and leaf extracts treated groups in low dose. However, High and medium dose of both extract and drug restores the disturbed pathological state in a highly significant manner (p>0.01) [Fig 2].

**Fig 3:**
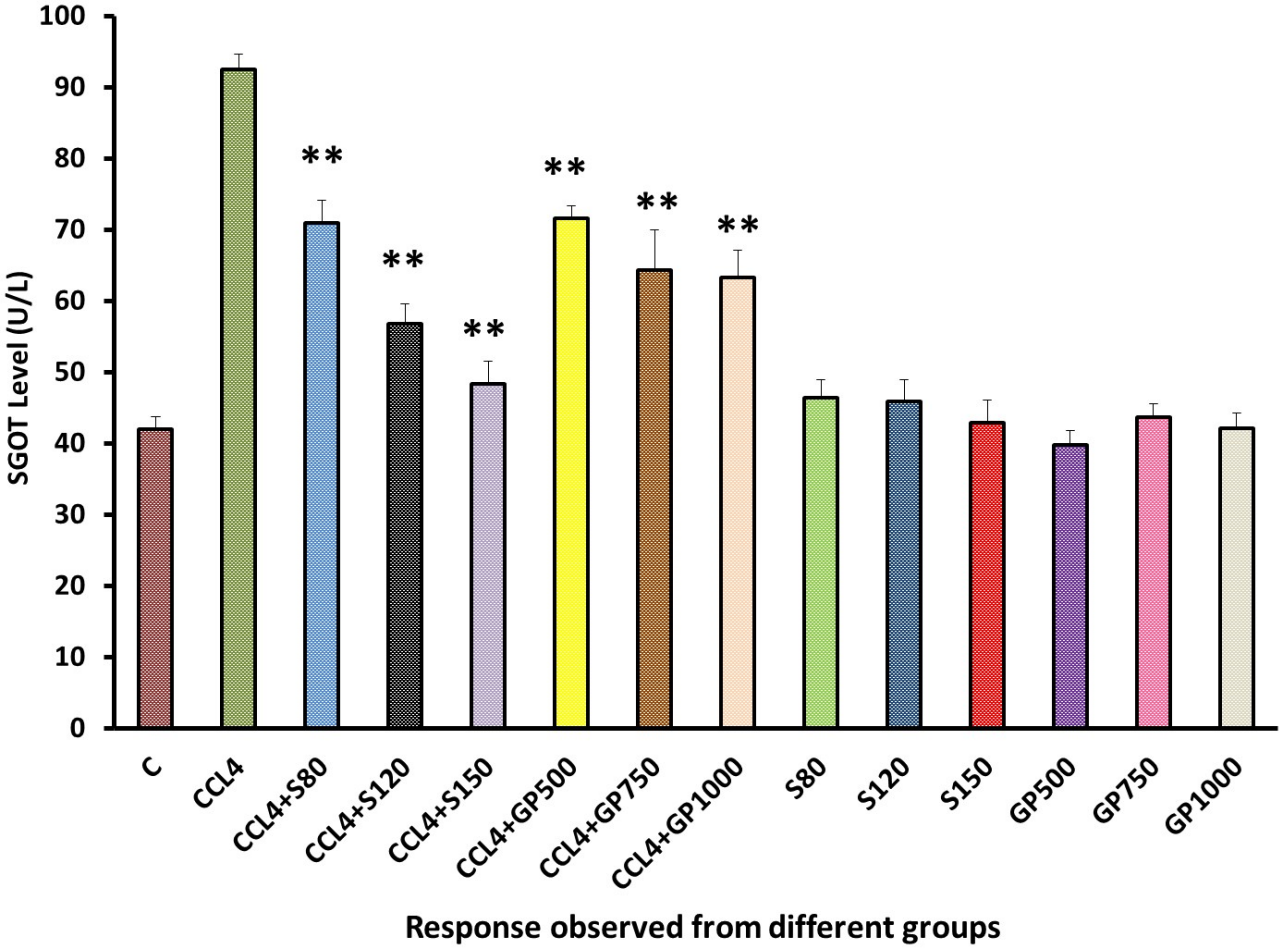
Effect of various treatment specimens on the SGOT level of CCl4- treated rats. Comparison of SGOT level (U/L) of rats, belonged to 14 groups just before sacrifice. Each group consist of 10 rodents each with equal body mass index. The data were expressed as mean±standard deviation. X axis represents the group distribution and y-axis represents SGOT level of different groups. All abbreviation of different groups has been mentioned in Table 1. (*indicates statistically significant change where p<0.05, correlation is significant at a 95% confidence interval and **indicates highly significant change where **p<0.01, correlation is significant at a 99% confidence interval).

SGOT level of positive control and disease control group showed two opposite scenarios [Fig 3]. Both plant extract and drug restore the disturbed pathological state in every single dose in a high significant manner (p>0.01), silymarin-treated hepatotoxic rats showed better results than *Gynura procumbens* treated rats.

**Fig 4:**
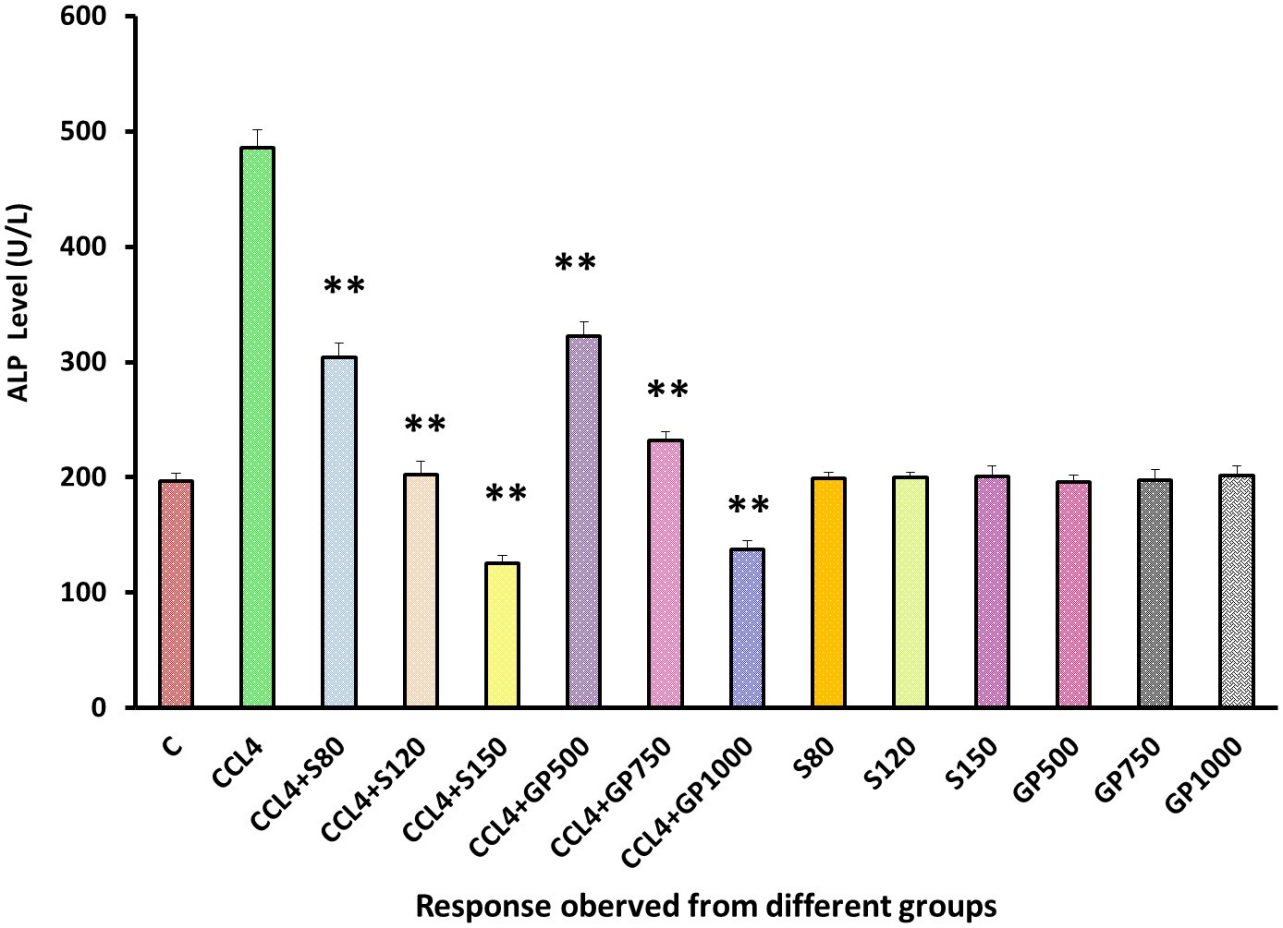
Effect of various treatment specimens on the ALP level of CCl4- treated rats. Comparison of ALP level (U/L) of rats, belonged to 14 groups just before sacrifice. Each group consist of 10 rodents each with equal body mass index. The data were expressed as mean±standard deviation. X axis represents the group distribution and y-axis represents ALP level of different groups. All abbreviation of different groups has been mentioned in Table 1. (*indicates statistically significant change where p<0.05, correlation is significant at a 95% confidence interval and **indicates highly significant change where p<0.01, correlation is significant at a 99% confidence interval).

Both drug and extracts showed statistical high significance (p<0.05) in ALP level reduction and ensured the effectiveness of *Gynura procumbens* extract [Fig 4]. No significant deviation from the negative control group in the ALP level yielded by the remaining six non-carbon tetrachloride-induced groups could be spotted.

**Fig 5:**
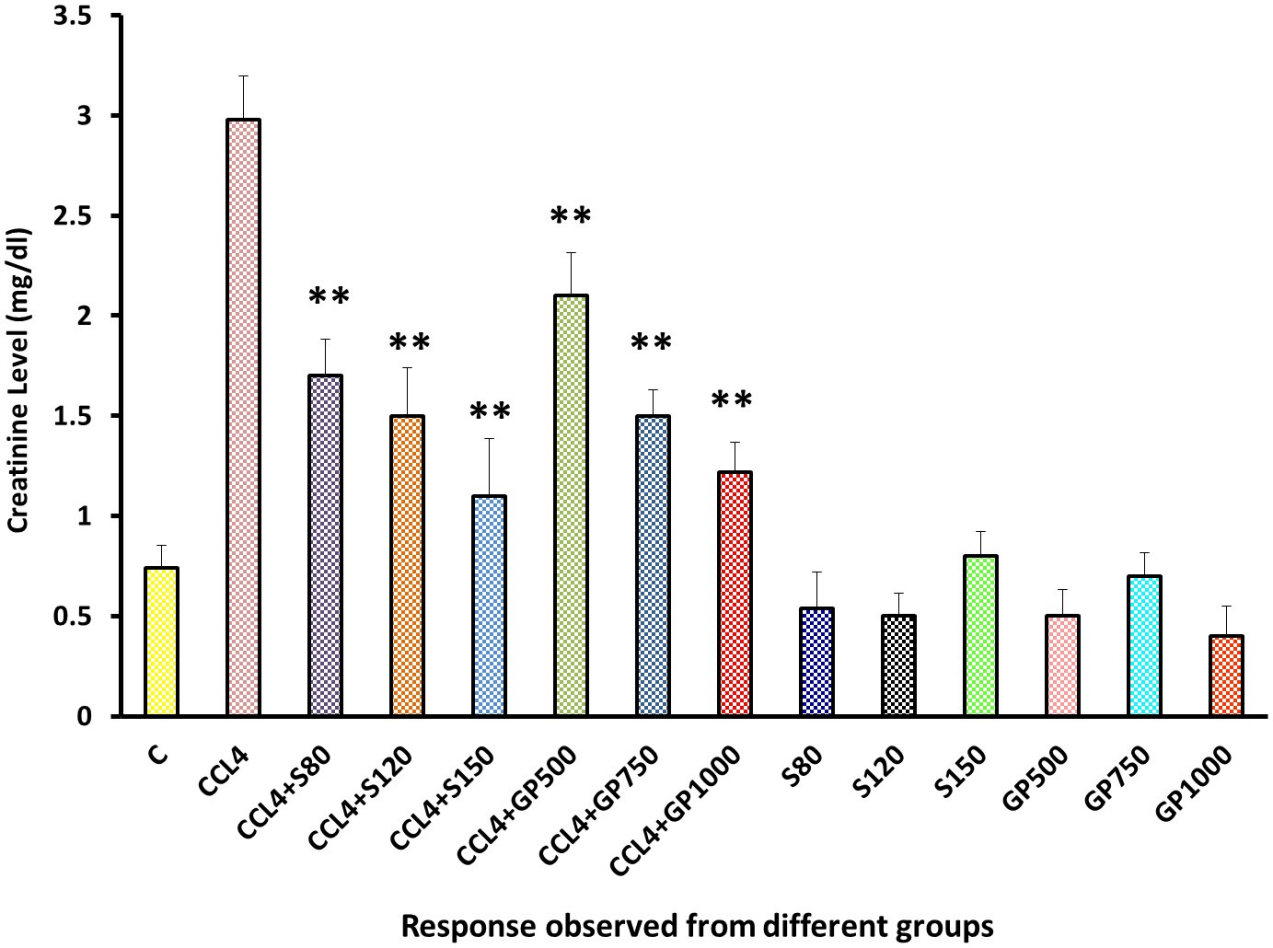
Effect of various treatment specimens on the creatinine level of CCl4- treated rats. Comparison of creatinine (mg/dl) level of rats, belonged to 14 groups just before sacrifice. Each group consist of 10 rodents each with equal body mass index. The data were expressed as mean±standard deviation. X axis represents the group distribution and y-axis represents creatinine level of different groups. All abbreviation of different groups has been mentioned in Table 1. (*indicates statistically significant change where p<0.05, correlation is significant at a 95% confidence interval and **indicates highly significant change where p<0.01, correlation is significant at a 99% confidence interval).

The disease control group displayed a significant increase in the creatinine level, which was in stark contrast with the negative control group. Silymarin and *Gynura procumbens* treatment gave noticeable results by reducing creatinine levels, and there was no significant rise of this with negative control groups [Fig 5].

**Fig 6:**
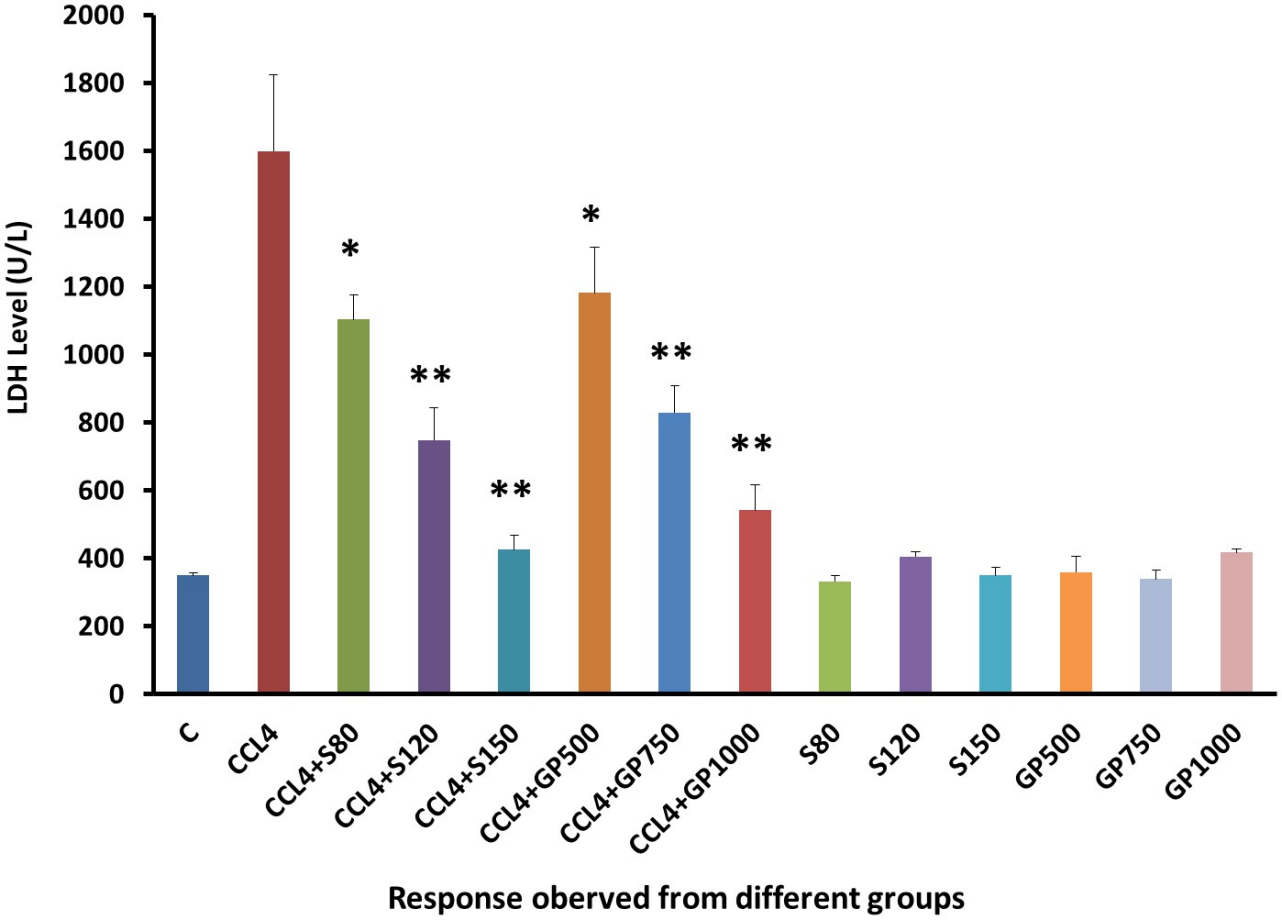
Effect of various treatment specimens on the LDH level of CCl4- treated rats. Comparison of LDH level (U/L) of rats, belonged to 14 groups just before sacrifice. Each group consists of 10 rodents each with equal body mass index. The data were expressed as mean±standard deviation. X-axis represents the group distribution and y-axis represents LDH level of different groups. All abbreviation of different groups has been mentioned in Table 1. (*indicates statistically significant change where p<0.05, correlation is significant at a 95% confidence interval and **indicates highly significant change where p<0.01, correlation is significant at a 99% confidence interval).

A four times increased LDH level was found in positive control group than that of negative control due to the devastating effect of CCL4. However, Low dose of treatment groups reduce the abnormally elevated LDH level successfully (p<0.05). Accordingly, Medium and High dose displayed a highly significant (p<0.05) reduction in LDH level [Fig 6].

**Fig 7:**
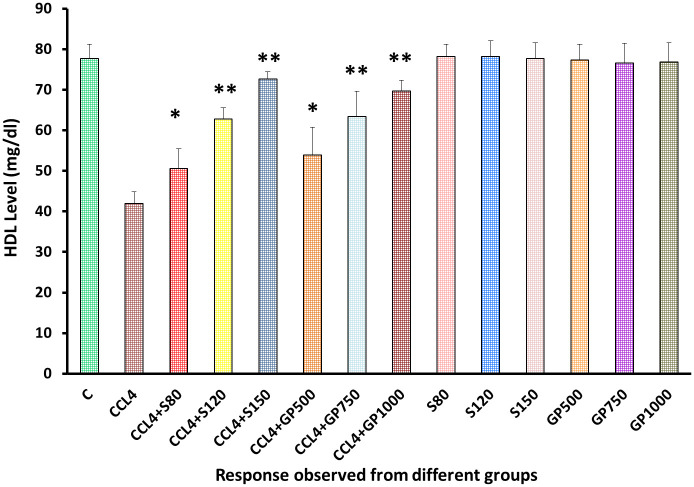
Effect of various treatment specimens on the HDL level of CCl4- treated rats. Comparison of HDL level (mg/dL) of rats, belonged to 14 groups just before sacrifice. Each group consist of 10 rodents each with equal body mass index. The data were expressed as mean±standard deviation. X-axis represents the group distribution and y-axis represents HDL level of different groups. All abbreviation of different groups has been mentioned in Table 1. (*indicates statistically significant change where p<0.05, correlation is significant at a 95% confidence interval and **indicates highly significant change where p<0.01, correlation is significant at a 99% confidence interval).

In the treatment groups, *Gynura procumbens* extract successfully enhance the diminished HDL level in a dose-dependent manner [Fig 7]. No severe observable effects were found upon the drug or plant extract administration to the non-CCl_4_ treated groups pointing to the safety of the plant extract.

**Fig 8:**
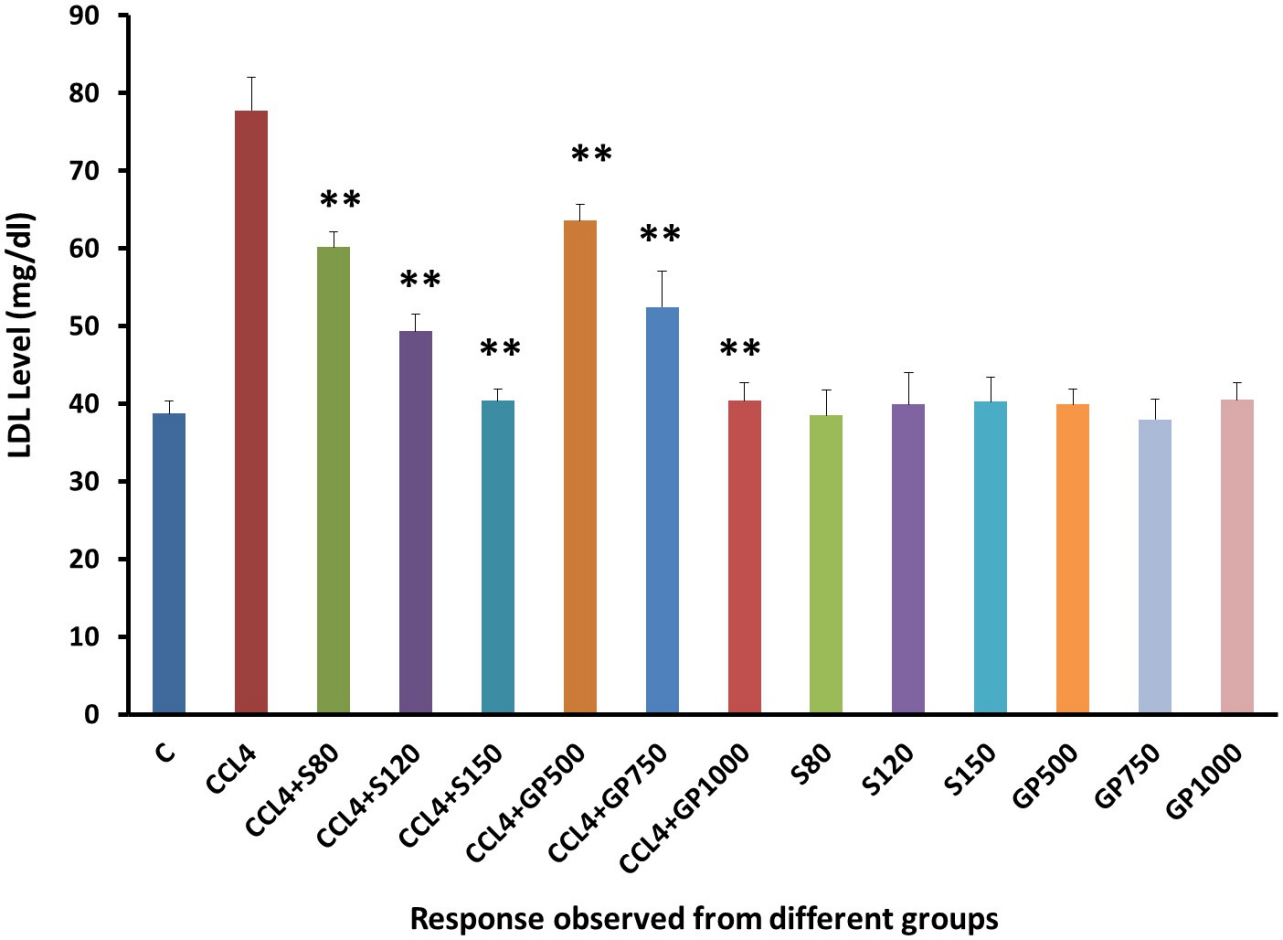
Effect of various treatment specimens on the LDL level of CCl4- treated rats. Comparison of LDL level (mg/dL) of rats, belonged to 14 groups just before sacrifice. Each group consist of 10 rodents each with equal body mass index. The data were expressed as mean±standard deviation. X-axis represents the group distribution and y-axis represents LDL level of different groups. All abbreviation of different groups has been mentioned in Table 1. (*indicates statistically significant change where p<0.05, correlation is significant at a 95% confidence interval and **indicates highly significant change where p<0.01, correlation is significant at a 99% confidence interval).

Compared to the negative control group, the disease control group yielded an increasing level of LDL following CCl_4_ administration [Fig 8]. Hepatic injured rats with the treatment of either Silymarin or *Gynura procumbens* extract with different doses overturned carbon tetrachloride mediated hike in LDL successfully.

**Fig 9:**
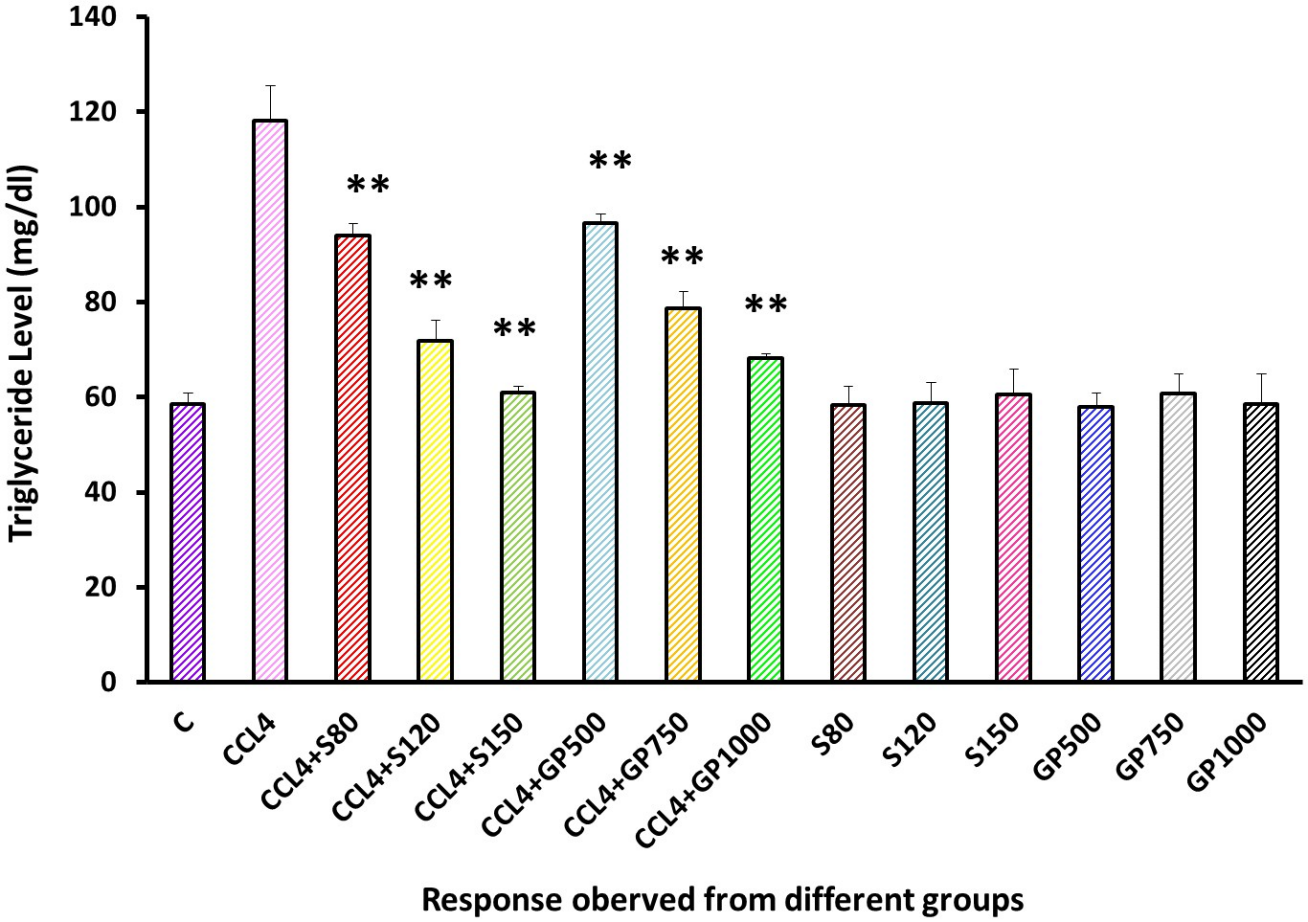
Effect of various treatment specimens on the Triglyceride level of CCl4- treated rats. Comparison of triglyceride (mg/dl) of rats, belonged to 14 groups just before sacrifice. Each group consist of 10 rodents each with equal body mass index. The data were expressed as mean±standard deviation. X-axis represents the group distribution and y-axis represents triglyceride level of different groups. All abbreviation of different groups has been mentioned in Table 1. (*indicates statistically significant change where p<0.05, correlation is significant at a 95% confidence interval and **indicates highly significant change where p<0.01, correlation is significant at a 99% confidence interval).

Triglyceride level was increased to almost double due to the application of carbon tetrachloride than the negative control group. However, this level went down to almost the same level as the treatment species when the highest dose was applied [Fig 9].

**Fig 10:**
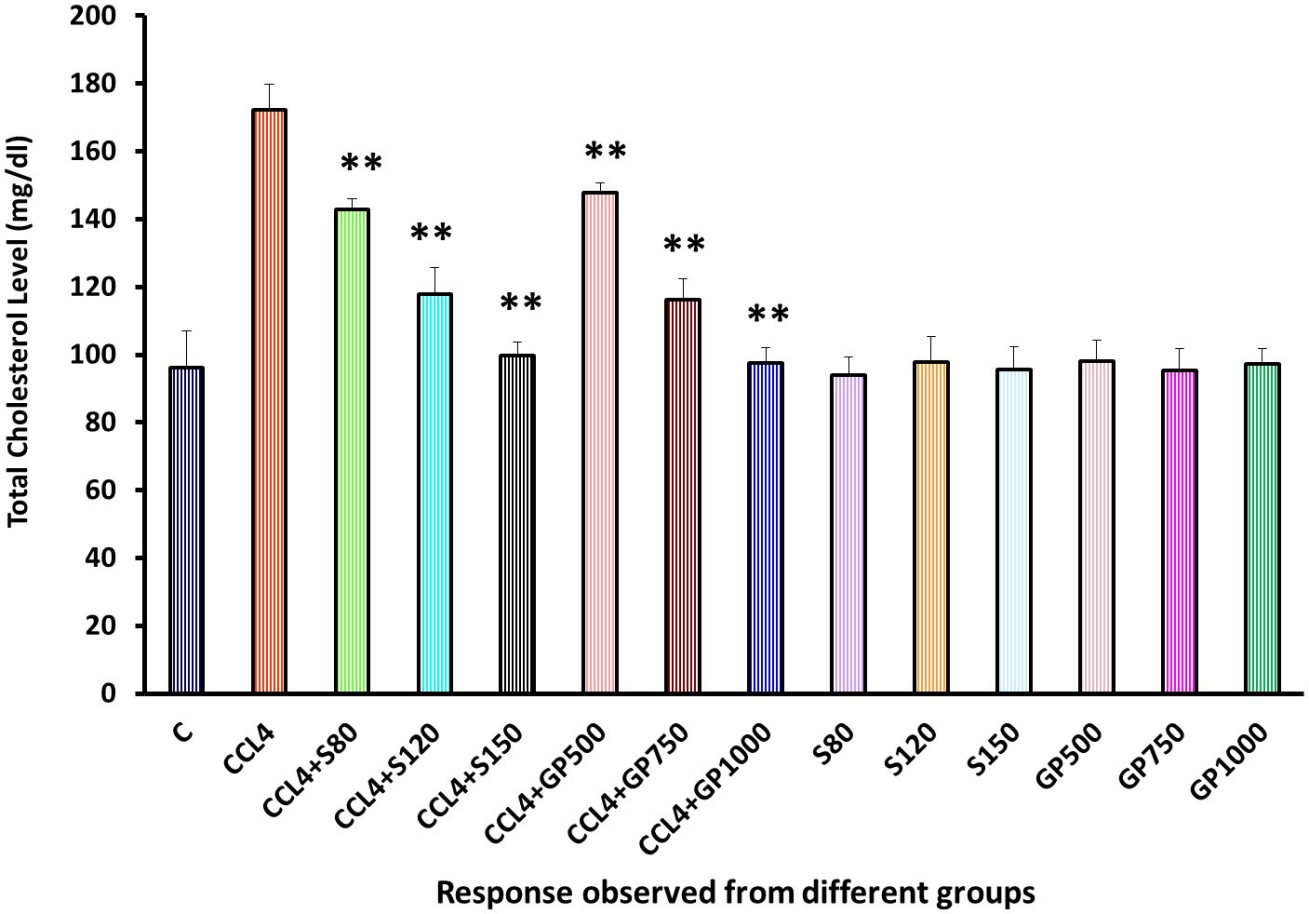
Effect of various treatment specimens on the total cholesterol level of CCl4- treated rats. Comparison of total cholesterol (mg/dl) level of rats, belonged to 14 groups just before sacrifice. Each group consist of 10 rodents each with equal body mass index. The data were expressed as mean±standard deviation. X-axis represents the group distribution and y-axis represents total cholesterol level of different groups. All abbreviation of different groups has been mentioned in Table 1. (*indicates statistically significant change where p<0.05, correlation is significant at a 95% confidence interval and **indicates highly significant change where p<0.01, correlation is significant at a 99% confidence interval).

Elevated cholesterol level was found in the disease control group [Fig 10]. On the contrary, both the drugs and extracts successfully reduced cholesterol levels in each treatment group (p<0.01). Supplementation of the highest dose showed the cholesterol level almost similar to the negative control group.

**Fig 11:**
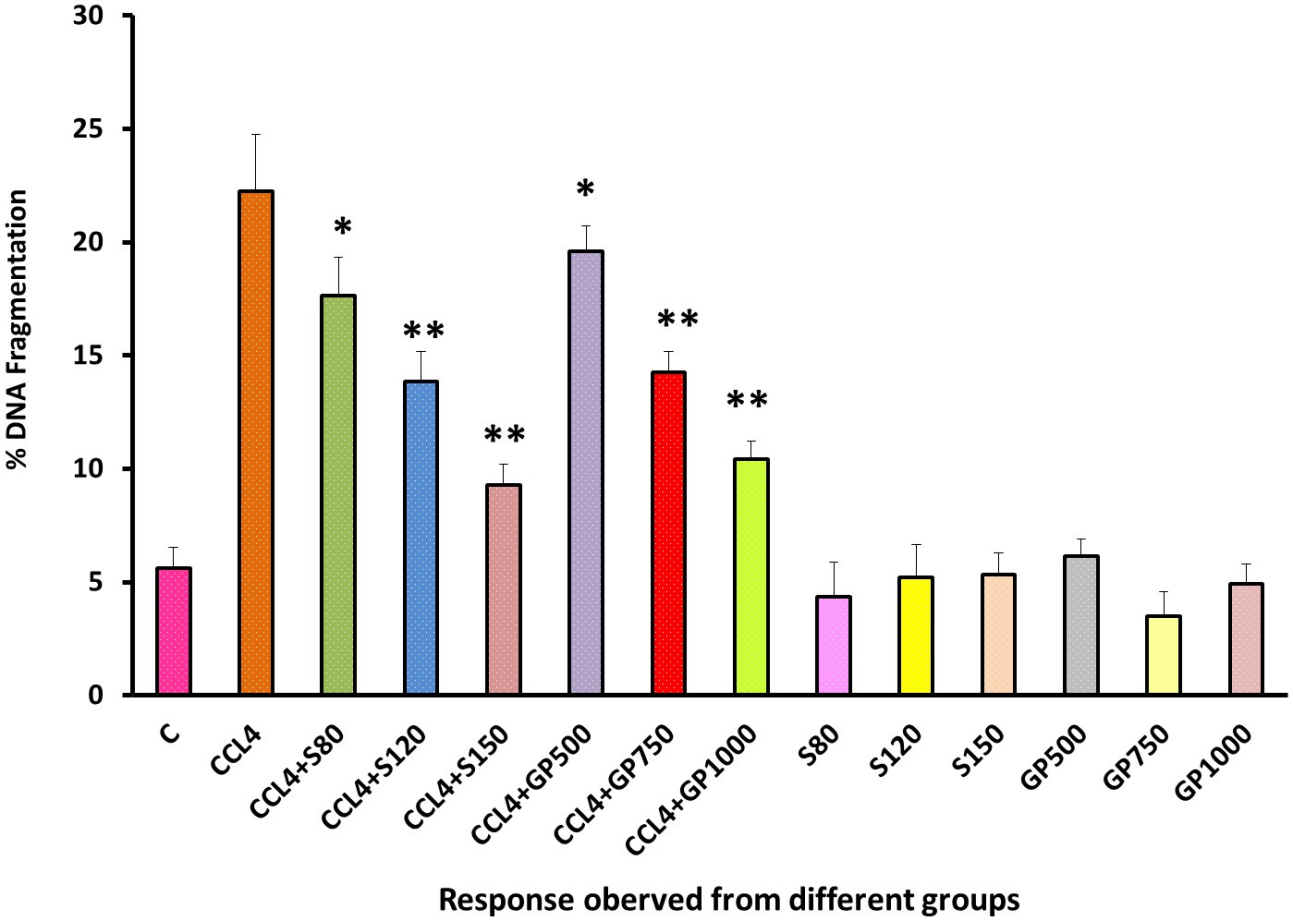
Effect of various treatment specimens on the DNA fragmentation of CCl4- treated rats. Comparison of DNA fragmentation level of rats, belonged to 14 groups just before sacrifice. Each group consist of 10 rodents each with equal body mass index. The data were expressed as mean±standard deviation. X-axis represents the group distribution and y-axis represents LDH level of different groups. All abbreviation of different groups has been mentioned in Table 1. (*indicates statistically significant change where p<0.05, correlation is significant at a 95% confidence interval and **indicates highly significant change where p<0.01, correlation is significant at a 99% confidence interval).

DNA fragmentation ranges were significantly (p<0.05) dropped by the low dose of silymarin and plant extracts applied to the treatment groups. Besides, the rate of DNA fragmentation reduced more precisely in medium dose and high dose that displayed a high significance difference when compared with positive control (p<0.05). At the same time, no significant changes were observed when treatment was performed on healthy groups to observe any changes in DNA [Fig 11].

**Fig 12:**
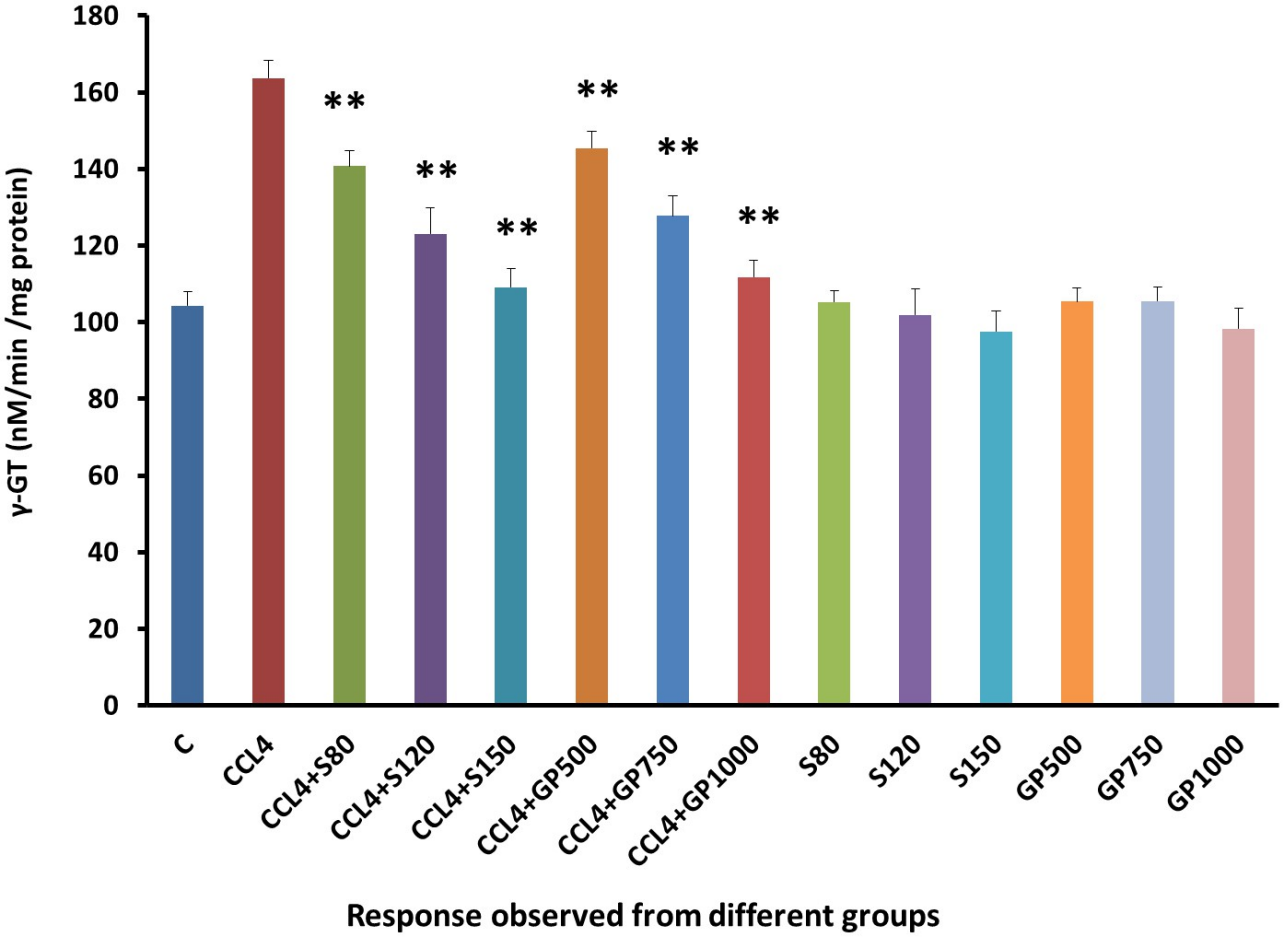
Effect of various treatment specimens on γ-GT levels of CCl4- treated rats. Comparison of γ-GT (nM/min/mg protein) level of rats, belonged to 14 groups just before sacrifice. Each group consist of 10 rodents each with equal body mass index. The data were expressed as mean±standard deviation. X-axis represents the group distribution and y-axis represents γ-GT level of different groups. All abbreviation of different groups has been mentioned in Table 1. (*indicates statistically significant change where p<0.05, correlation is significant at a 95% confidence interval and **indicates highly significant change where p<0.01, correlation is significant at a 99% confidence interval).

γ-GT levels were increased abnormally in group 2 due to the destructive effect of CCL4. Alongside, γ-GT levels decreased in all treatment groups in a highly significant (p<0.01) approach in comparison to the disease control Group [Fig 12].

**Fig 13:**
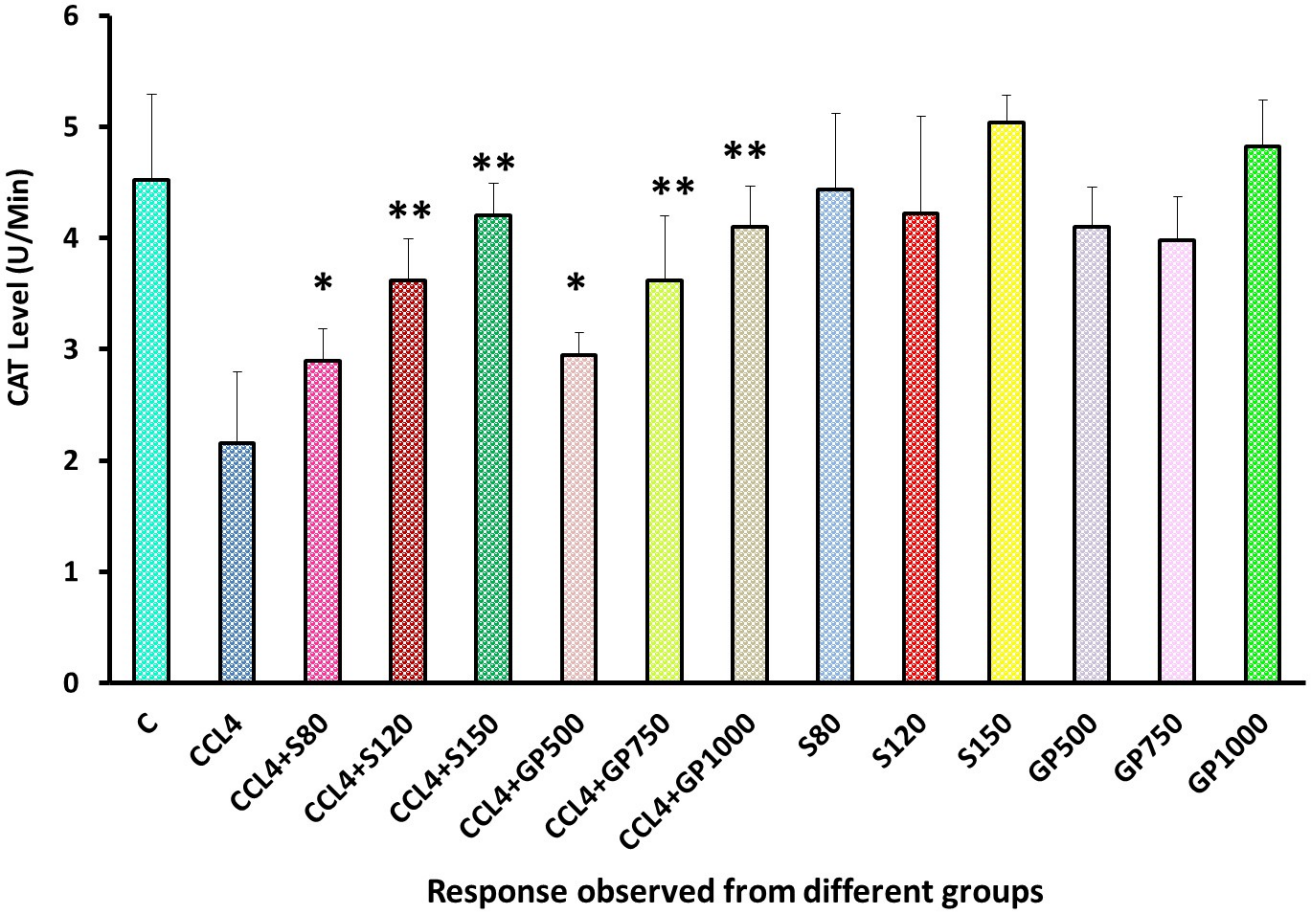
Effect of various treatment specimens on CAT levels of CCl4- treated rats. Comparison of CAT (U/Min) level of rats belonging to 14 groups just before sacrifice. Each group consist of 10 rodents each with equal body mass index. The data were expressed as mean±standard deviation. X-axis represents the group distribution and y-axis represents CAT level of different groups. All abbreviation of different groups has been mentioned in Table 1. (*indicates statistically significant change where p<0.05, correlation is significant at a 95% confidence interval and **indicates highly significant change where p<0.01, correlation is significant at a 99% confidence interval).

A noticeable difference was observed between the negative control and disease control groups in the CAT level, as the reduction of the CAT level was devastating after supplying carbon tetrachloride to the rats. On the other hand, both treatment groups of silymarin or plant extracts experienced a highly significant (p<0.01) reduction in CAT level [Fig 13].

**Fig 14:**
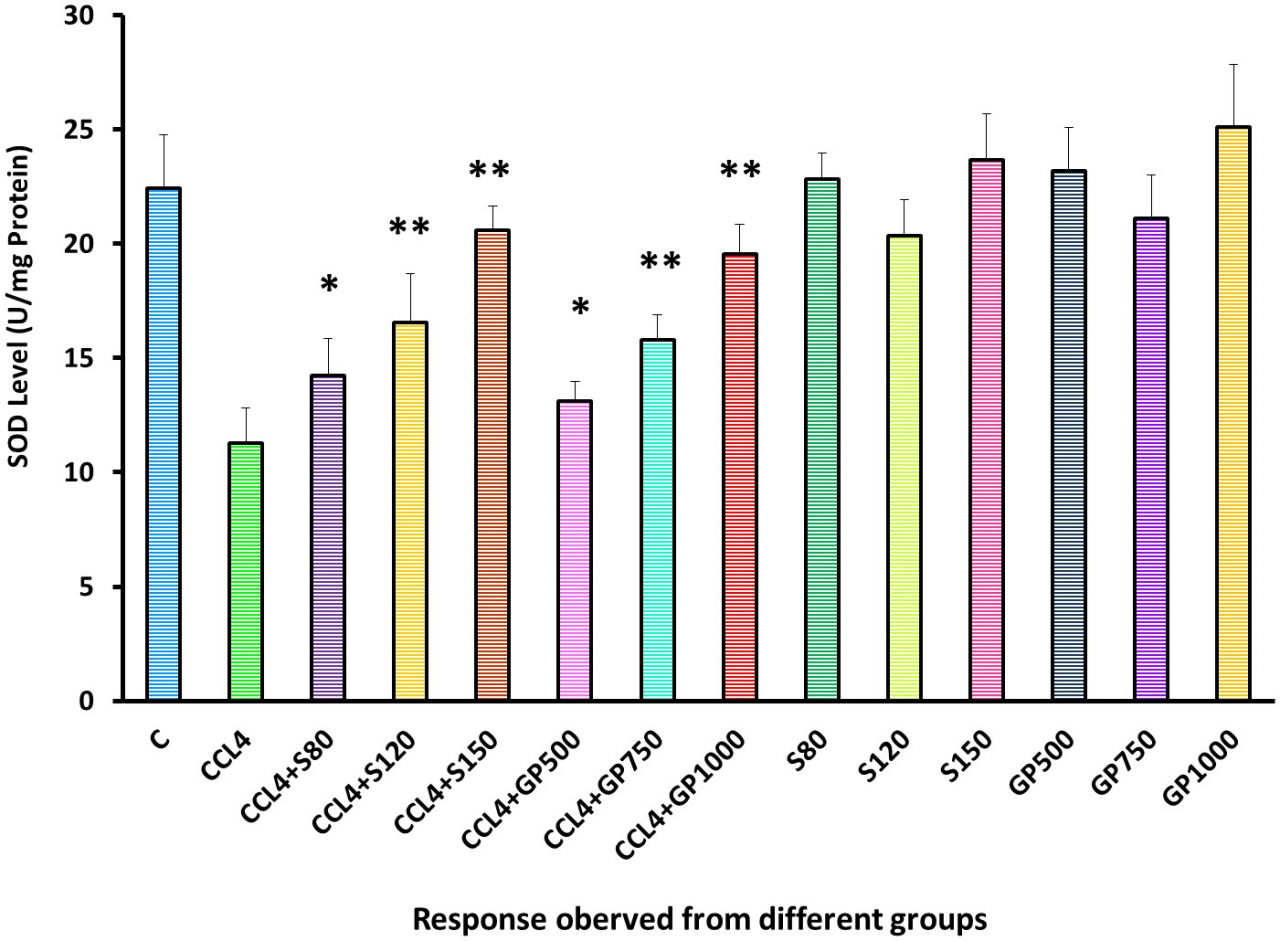
Effect of various treatment specimens on SOD levels of CCl4- treated rats. Comparison of SOD (U/mg Protein) level of rats, belonged to 14 groups just before sacrifice. Each group consist of 10 rodents each with equal body mass index. The data were expressed as mean±standard deviation. X-axis represents the group distribution and y-axis represents SOD level of different groups. All abbreviation of different groups has been mentioned in Table 1. (*indicates statistically significant change where p<0.05, correlation is significant at a 95% confidence interval and **indicates highly significant change where p<0.01, correlation is significant at a 99% confidence interval).

In the Superoxide dismutase test, the disease control group demonstrated a remarkable turn down of this level, indicating the rodents’ decreased antioxidant defense. However, statistically significant improvement (P<0.05) was observed in the low dose of treatment groups of silymarin and *Gynura procumbens*. Consequently, a high significant (p<0.01) enhancement of SOD level were experienced in medium and high dose treatment of both drug and plant treated group [Fig 14].

**Fig 15:**
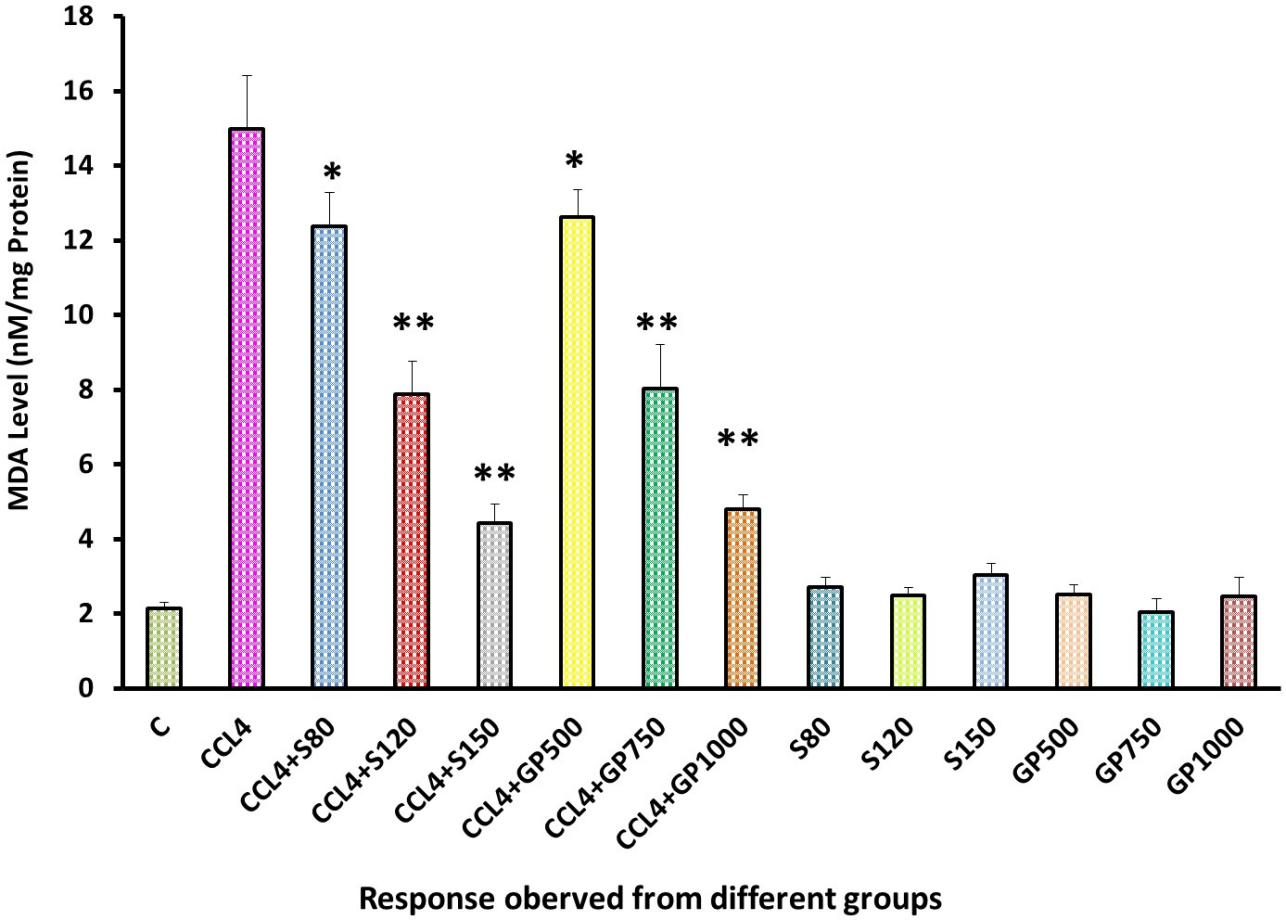
Effect of various treatment specimens on MDA levels of CCl4- treated rats. Comparison of MDA (nM/mg Protein) level of rats, belonged to 14 groups just before sacrifice. Each group consist of 10 rodents each with equal body mass index. The data were expressed as mean±standard deviation. X-axis represents the group distribution and y-axis represents MDA level of different groups. All abbreviation of different groups has been mentioned in Table 1. (*indicates statistically significant change where p<0.05, correlation is significant at a 95% confidence interval and **indicates highly significant change where p<0.01, correlation is significant at a 99% confidence interval).

A significant overproduction of malondialdehyde was noticed in the diseased group. After administering the test extract and the standard drug, a significant statistical difference (p<0.05) was detected in the low dose of treatment groups as the MDA level rate decreased. Besides, medium dose and high dose of both extract and drug restore the MDA level in a highly significant (p<0.01) approach [Fig 15].

### Results of histopathological study

**Fig 16:**
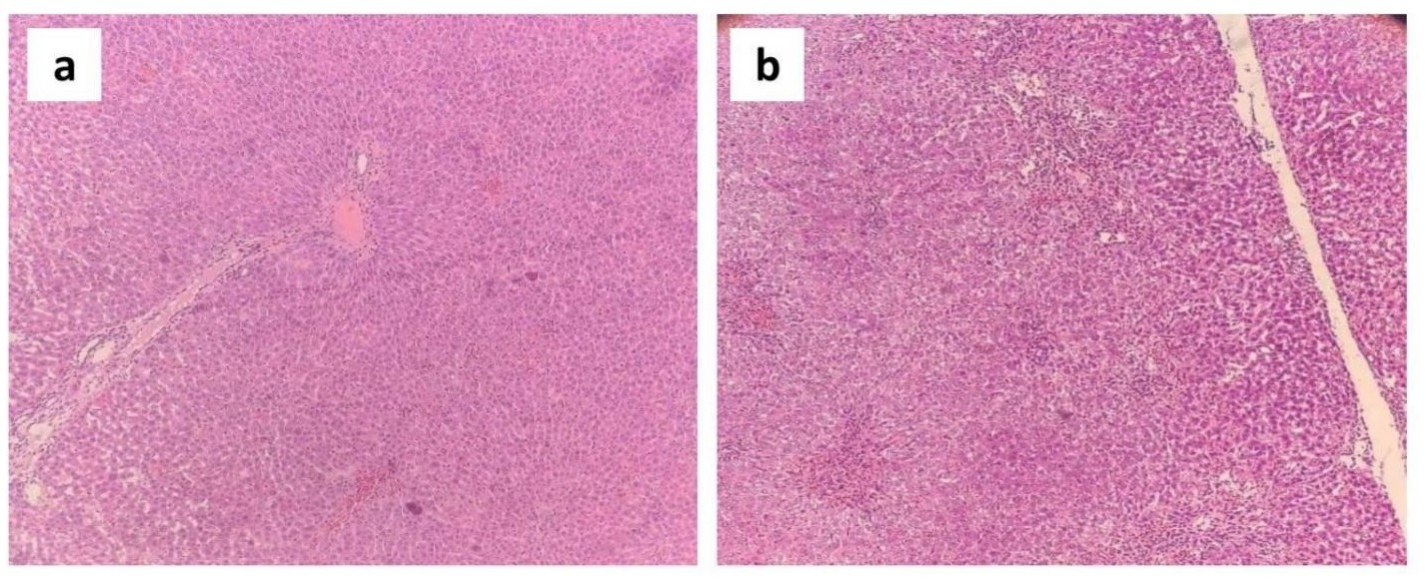
Photomicrographs (10×) of histopathological analysis of liver samples taken from the (a) negative control group and (b) disease control group.

The histoarchitecture of the negative control group exhibited normal liver cells with sound hepatocytes, distinct sinusoidal spaces, well-defined lobular units, and portal tracts (Fig 16a). In contrast, in the disease control group, the histoarchitecture of the liver samples showed significant hepatocellular swelling with intense lobular and portal tract inflammation, severe microvesicular steatosis, and sinusoidal dilation (Fig 16b). These features have been portrayed in Fig 16.

**Fig 17:**
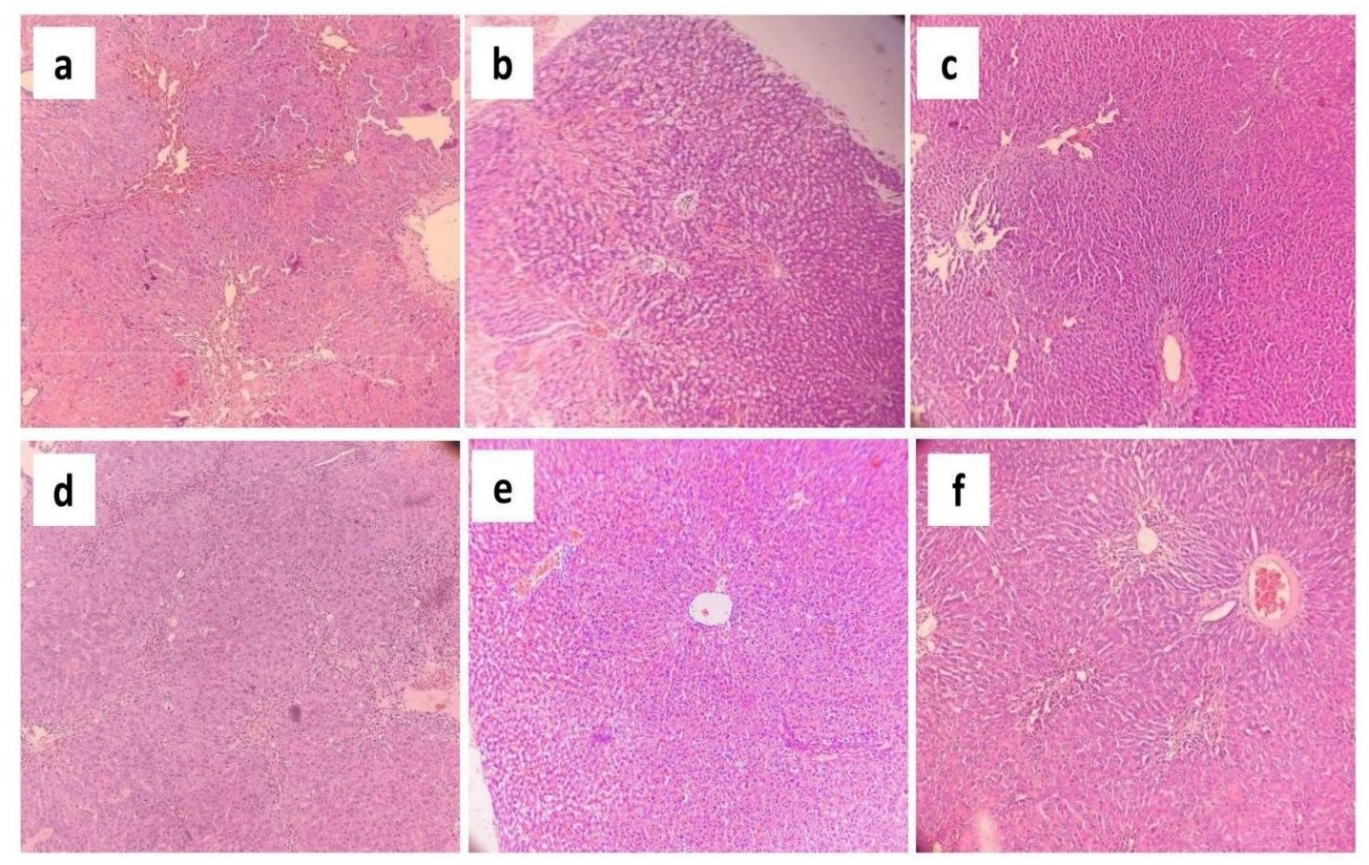
Photomicrographs (10×) of histopathological analysis of liver samples taken from the experimental rats. (a) Liver architecture of rats treated with low dose of silymarin. (b) Liver architecture of rats treated with medium dose of silymarin. (c) Liver architecture of rats treated with high dose of silymarin. (d) Liver architecture of rats treated with low dose of *G*. *procumbens*. (e) Liver architecture of rats treated with medium dose of *G*. *procumbens*. (f) Liver architecture of rats treated with high dose of *G*. *procumbens*.

The histopathological changes of the disease control group were almost entirely reversed by administering varying doses (i.e. low, medium and high doses) of the standard drug, silymarin (Fig 17–17c). However, administration of a high dose of *G*. *procumbens* to the disease control group also showed a dramatic reversal of these CCl4-induced histopathological alterations, illustrating very mild inflammation in the portal tract and slight dilatation of sinusoids (Fig 17f). But this reversal was a little bit weaker compared to the standard drug. On the other hand, treatment with a medium dose of *G*. *procumbens* exerted moderate reversal of CCl_4_ mediated histopathological alterations, exhibiting mild microvesicular steatosis, moderate portal inflammation, and sinusoidal dilatation (Fig 17e), while a low dose of *G*. *procumbens* treatment displayed quite negligible reversal of the CCl_4_ induced histopathological changes in contrast to the standard drug (Fig 17d). These changes have been illustrated in Fig 17.

The histopathological changes observed in different experimental groups have been summarized in Table 2.

**Table 2:** Histopathological alterations in experimental rats.

Histopathological Changes	Group Status							
	Negative Control	Disease Control	CCl4+Low dose Silymarin	CCl4+Medium dose Silymarin	CCl4+High dose Silymarin	CCl4+Low dose *G*. *procumbens*	CCl4+Medium dose *G*. *procumbens*	CCl4+High dose *G*. *procumbens*
Swelling of the hepatocytes	—	√	—	—	—	—	—	—
Cholestasis	—	√	—	—	—	—	—	—
Hepatocellular necrosis	—	—	—	—	—	—	—	—
Microvesicular Steatosis	—	√	—	—	—	√	√	—
Macrovesicular steatosis	—	√	—	—	—	√	—	—
Lobular inflammation	—	√	√	—	—	—	—	—
Portal inflammation	—	√	√	—	—	√	√	—
Fibrosis	—	—	—	—	—	—	—	—
Dilatation of sinusoids	—	√	—	—	—	√	√	—

“√“ denotes presence of a particular histopathological feature and “—" denotes absence of particular histopathological feature.

### Results of *in silico* study

Of the 76 phytoconstituents of *G*. *procumbens*, 11 were alkaloids, 32 were phenolic compounds, 13 were flavonoids, 15 were steroidal compounds, and 5 were terpenes [78]. The compounds with their respective compound IDs (to be used henceforth in this manuscript) are presented in Table 3.

**Table 3:** Phytoconstituents of *G*. *procumbens*.

Types	Constituents	Compound code
Alkaloids	Erucifoline-N-oxide	C_1
	Heliotrine	C_2
	Lasiocarpine	C_3
	Lycopsamine-N-oxide	C_4
	Retronecine	C_5
	Retrorsine	C_6
	Senecionine-N-oxide	C_7
	Seneciphylline	C_8
	Seneciphylline-N-oxide	C_9
	Senecivernine	C_10
	Senkirkine	C_11
Phenolic Compounds	1, 4-Dicaffeoylquinic acid	C_12
	1, 5-Dicaffeoylquinic acid	C_13
	3, 4-Dicaffeoylquinic acid	C_14
	3, 4-dicaffeoylquinic acid methyl ester	C_15
	3, 5-Dicaffeoylquinic acid	C_16
	3, 5-dicaffeoylquinic acid ethyl ester	C_17
	3, 5-dicaffeoylquinic acid methyl ester	C_18
	3-Caffeoylquinic acid	C_19
	4, 5-Dicaffeoylquinic acid	C_20
	4, 5-dicaffeoylquinic acid methyl ester	C_21
	4-Caffeoylquinic acid	C_22
	Caffeic acid	C_23
	Caffeoylglucaric acid	C_24
	Chlorogenic acid	C_25
	cis-5-p-Coumaroylquinic acid	C_26
	Coumaric acid glucoside	C_27
	Ferulic acid	C_28
	Feruloylquinic acid	C_29
	Gallic acid	C_30
	Gallic acid glucoside	C_31
	Hydroxybenzoic acid glucoside	C_32
	Hydroxytyrosol glucoside	C_33
	Neochlorogenic acid	C_34
	p-Coumaric acid	C_35
	p-Hydroxybenzoic acid	C_36
	Protocatechuic acid	C_37
	Protocatechuic acid glucoside	C_38
	Sinapic acid	C_39
	Sinapic acid glucoside	C_40
	Syringic acid	C_41
	trans-5-p-Coumaroylquinic acid	C_42
	Vanillic acid	C_43
Flavonoid Compounds	Apigenin	C_44
	Astragalin (Kaempferol-3-O-β-D glucoside)	C_45
	Eriocitrin	C_46
	Homoorientin	C_47
	Kaempferol	C_48
	Kaempferol-5-O-(6″-O-acetyl)-β-D-glucopyranoside	C_49
	Luteolin	C_50
	Myricetin	C_51
	Negletein	C_52
	Nicotiflorin (Kaempferol-3-O-rutinoside/ Kaempferol-3-O-rhamnosyl-1→6-glucoside)	C_53
	Quercetin	C_54
	Quercetin 3-O-rhamnosyl-1→2-galactoside	C_55
	Rutin (Quercetin-3-O-rutinoside/Quercetin-3-O-rhamnosyl-1→6- glucoside)	C_56
Steroidal Compounds	3-O-β-D-Glucopyranosyl stigmasterol	C_57
	Daucosterol (3-O-β-D-Glucopyranosyl-β-sitosterol)	C_58
	β-Sitosterol	C_59
	β-Stigmasterol	C_60
	3-carene	C_61
	4β, 10α-Aromadendranediol	C_62
	Bicyclo[4.1.0]hept-2-ene	C_63
	Caryophyllene	C_64
	Limonene	C_65
	Muurol-4-ene-1β, 3β, 10βtriol	C_66
	Muurol-4-ene-1β, 3β, 10β-triol 3-O-β-D-glucopyranoside	C_67
	Muurol-4-ene-1β, 3β, 15-triol 3-O-β-Glucopyranoside	C_68
	Negunfurol	C_69
	Schensianol A	C_70
	α-phellandrene	C_71
Terpenes	α-pinene	C_72
	β-Caryophyllene	C_73
	β-Humulene	C_74
	β-myrcene	C_75
	β-pinene	C_76

The macromolecule target receptors, their binding sites, and their respective controls are presented in Table 4.

**Table 4:** Macromolecular targets for hepatoprotective activity.

Macromolecule Name	PDB ID	Control drug	Vina Search Space		Reference
			Center	Dimensions (Angstrom)	
TGF-β Type I Receptor	1VJY	Galunisertib	X: 11.8856	X: 20.4315	[7, 8]
			Y: 66.3307	Y: 20.1017	
			Z: 4.7346	Z: 18.2275	
PPAR-α	5HYK	Bezafibrate	X: 12.1827	X: 17.2809	[9, 10]
			Y: 23.8565	Y: 12.1112	
			Z: 25.1561	Z: 15.6780	

**Fig 18:**
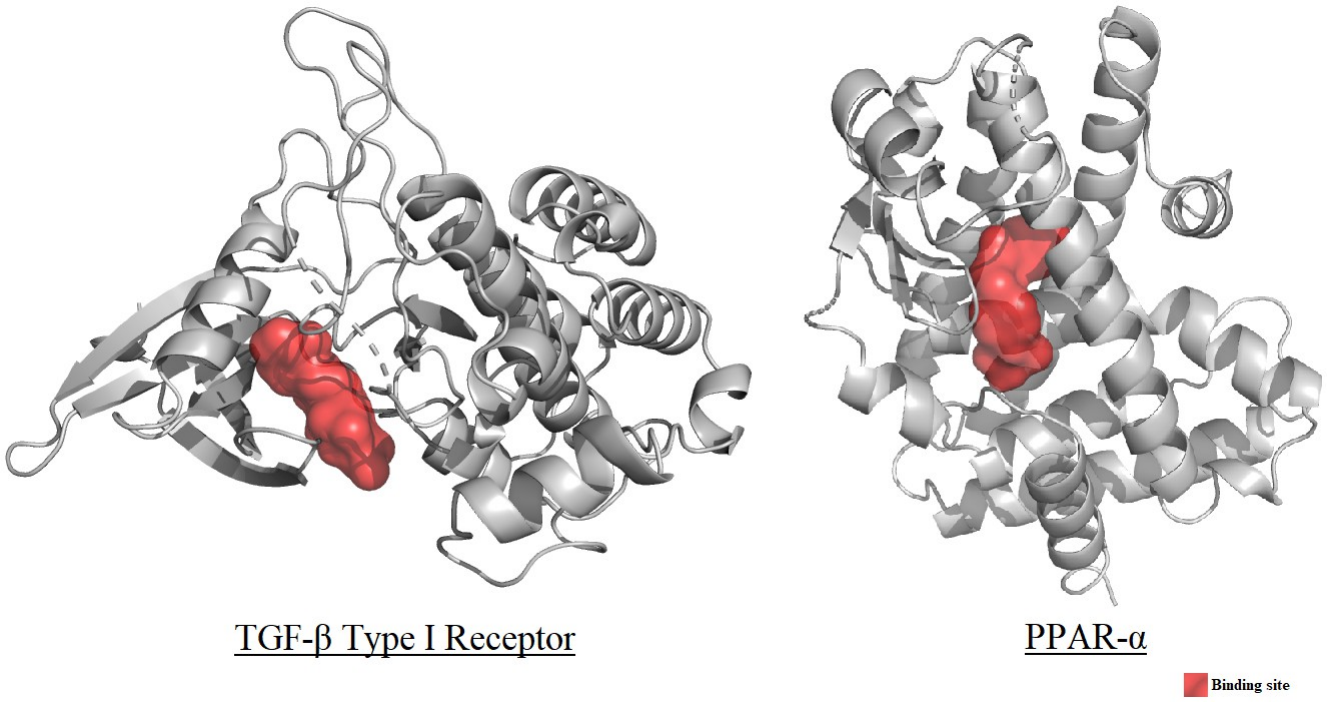
Binding sites of macromolecules TGF-β type I receptor and PPAR-α (binding site marked in red).

The target macromolecular receptors and their binding sites have been illustrated in Fig 18.

**Fig 19:**
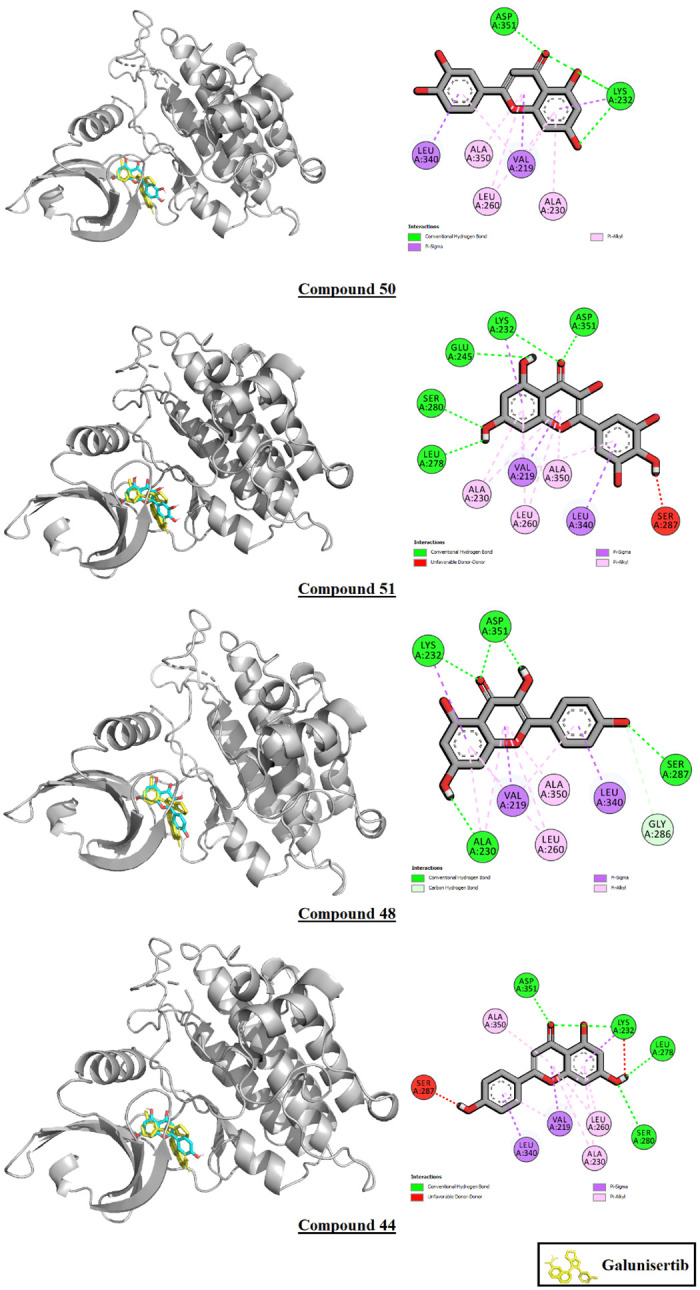
Interactions of compounds 44, 48, 50, and 51 with TGF-β type I receptor.

All 76 phytoconstituents assayed *in silico* displayed lower binding affinity values than the control galunisertib (B.A.: -11.5 Kcal/mole) against the TGF-β type I receptor. Compound 50, or luteolin, displayed the highest binding affinity after the control at -10.3 Kcal/mole, followed by compound 51 (myricetin) at -10.2 Kcal/mole. Of the top 10 highest binding affinity phytoconstituents, 7 were flavonoids, while the rest were phenolic compounds. All phytoconstituents shared interaction residues with the control. The interactions of the top 10 binding affinity phytoconstituents with the TGF-β type I receptor are demonstrated in Table 5 and Fig 19.

**Table 5:** Interactions of the top 10 binding affinity phytoconstituents with TGF-β type I receptor.

Ligand	Binding affinity (Kcal/mole)	Ligand-Macromolecule interactions
Galunisertib (control)	-11.5	ILE A:211*, VAL A:219*, ALA A:230*, LYS A:232*, TYR A:249*, LEU A:260*, LEU A:278*, ASP A:281*, TYR A:282*, HIS A:283*, LEU A:340*, ASP A:351*
C_50	-10.3	VAL A:219*, ALA A:230*, LYS A:232*, LEU A:260*, LEU A:340*, ALA A:350, ASP A:351*
C_51	-10.2	VAL A:219*, ALA A:230*, LYS A:232*, GLU A:245, LEU A:260*, LEU A:278*, SER A:280, SER A:287, LEU A:340*, ALA A:350, ASP A:351*
C_20	-10.1	LYS A:213, ALA A:230*, LYS A:232*, LEU A:260*, HIS A:283*, LEU A:340*
C_44	-10.1	VAL A:219*, ALA A:230*, LYS A:232*, LEU A:260*, LEU A:278*, SER A:280, SER A:287, LEU A:340*, ALA A:350, ASP A:351*
C_48	-10.1	VAL A:219*, ALA A:230*, LYS A:232*, LEU A:260*, GLY A:286, SER A:287, LEU A:340*, ALA A:350, ASP A:351*
C_13	-10	ILE A:211*, GLY A:214, ALA A:230*, LYS A:232*, LEU A:260*, HIS A:283*, SER A:287, LYS A:337, ASP A:351*
C_54	-10	VAL A:219*, ALA A:230*, LYS A:232*, GLU A:245, LEU A:260*, LEU A:278*, SER A:280, GLY A:286, LEU A:340*, ALA A:350, ASP A:351*
C_45	-9.7	ILE A:211*, VAL A:219*, ALA A:230*, LYS A:232*, LEU A:260*, TYR A:282*, SER A:287, ASP A:290, LYS A:337, LEU A:340*, ALA A:350, ASP A:351*
C_52	-9.7	ILE A:211*, VAL A:219*, ALA A:230*, LYS A:232*, LEU A:260*, LEU A:278*, LEU A:340*, ALA A:350, ASP A:351*
C_16	-9.6	ILE A:211*, VAL A:219*, LYS A:232*, LEU A:260*, HIS A:283*, SER A:287, ASN A:338, LEU A:340*

Interactions common with the control*

**Fig 20:**
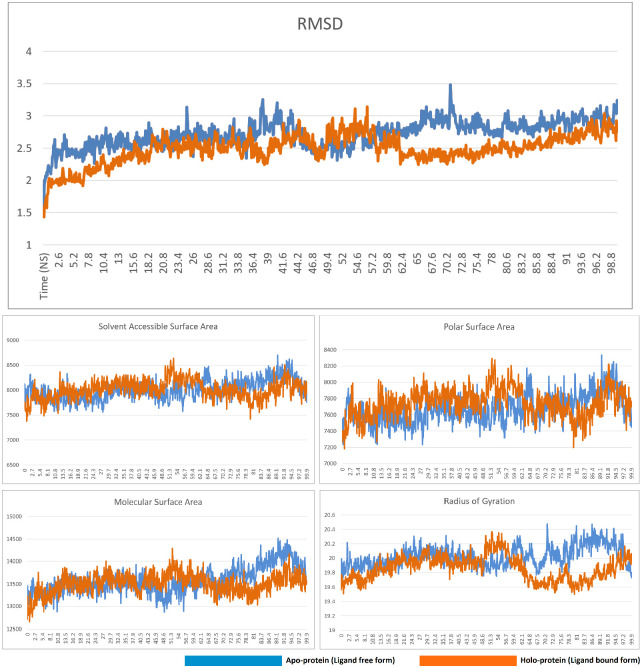
RMSD, solvent accessible surface area, polar surface area, molecular surface area, and radius of gyration of the free and compound 50-bound form of TGF-β type I receptor.

In the molecular dynamics simulation study, the complex of compound 50 and TGF-β type I receptor was observed to be stable. The RMSD, solvent accessible surface area, polar surface area, and molecular surface area of the ligand-bound form closely resembled the data from the non-ligand bound apo form of the receptor, indicating that the binding of the ligand did not result in any loss of stability. The radius of gyration data also closely paralled the apo form until the 62^nd^ nanosecond, after which the ligand-bound displayed a slightly lower radius of gyration than the non-ligand bound apop form. Overall, the data clearly indicated that the complex was stable. The RMSD, solvent accessible surface area, polar surface area, molecular surface area, and radius of gyration of the free and compound 50-bound form of TGF-β type I receptor is presented in Fig 20.

**Fig 21:**
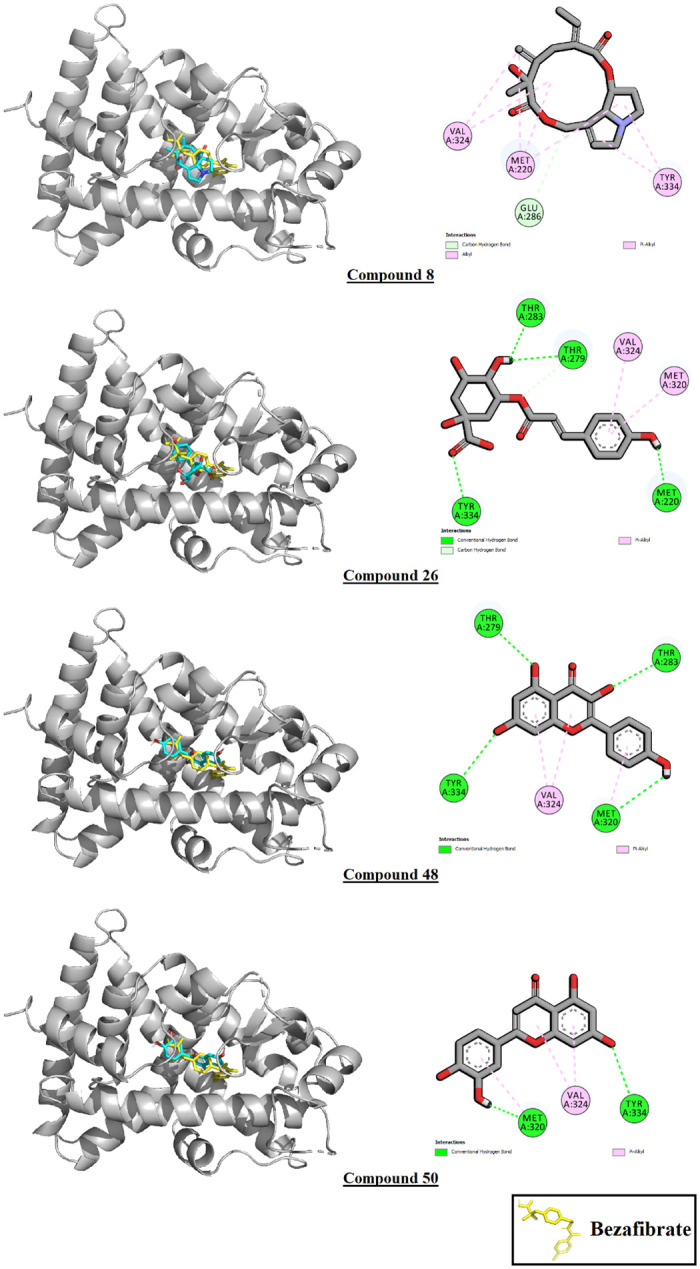
Interactions of compounds 8, 26, 48 and 50 with PPAR-α.

26 phytoconstituents out of 76 displayed higher binding affinity values than the control bezafibrate (B.A.: -7.6 Kcal/mole) against PPAR-α. Compound 8, or seneciphylline displayed the highest binding affinity after the control at -8.7 Kcal/mole, followed by compound 26 (cis-5-p-coumaroylquinic acid) at -8.6 Kcal/mole and compound 27 (coumaric acid glucoside) at -8.5 Kcal/mole. Of these 26, 15 were phenolic compounds, 6 were flavonoids, 2 were steroidal compounds, 2 were terpenes, and 1 was an alkaloid. Of the top 10 highest binding affinity phytoconstituents, 1 was an alkaloid, 6 were flavonoids, and 3 were phenolic compounds. All phytoconstituents except compounds 25, 26, 34, and 38 shared interaction residues with the control. The interactions of the top 10 binding affinity phytoconstituents with the receptor PPAR-α are demonstrated in Table 6. The interactions of the 4 top scoring ligands have been illustrated in Fig 21.

**Table 6:** Interactions of the top 10 binding affinity phytoconstituents with PPAR-α.

Ligand	Binding affinity (Kcal/mole)	Ligand-macromolecule interactions
Bezafibrate (control)	-7.6	MET A:220*, CYS A:276*, TYR A:314*, ILE A:317*, PHE A:318*, LEU A:321*, VAL A:324*, LEU A:331*
C_8	-8.7	GLU A:286, MET A:220*, VAL A:324*, TYR A:334
C_26	-8.6	TYR A:334, THR A:283, THR A:279, MET A:320
C_27	-8.5	SER A:323, MET A:220*, VAL A:332, TYR A:334, LEU A:321*, VAL A:324*, TYR A:214, THR A:283
C_48	-8.5	THR A:283, TYR A:334, THR A:279, VAL A:324*, MET A:320
C_50	-8.4	TYR A:334, THR A:279, VAL A:324*, MET A:320
C_54	-8.4	THR A:283, TYR A:214, VAL A:324*, MET A:320
C_15	-8.3	THR A:283, GLU A:286, MET A:320, LEU A:321*, CYS A:276*, MET A:220*, TYR A:334
C_44	-8.3	TYR A:334, TYR A:214, MET A:220*, THR A:279, MET A:320, VAL A:324*
C_51	-8.3	THR A:283, GLU A:286, THR A:279, LEU A:321*, ASN A:219, MET A:220*
C_31	-8.2	THR A:283, TYR A:334, GLU A:286, MET A:220*, THR A:279, SER A:323, MET A:320

Interactions common with the control*

**Fig 22:**
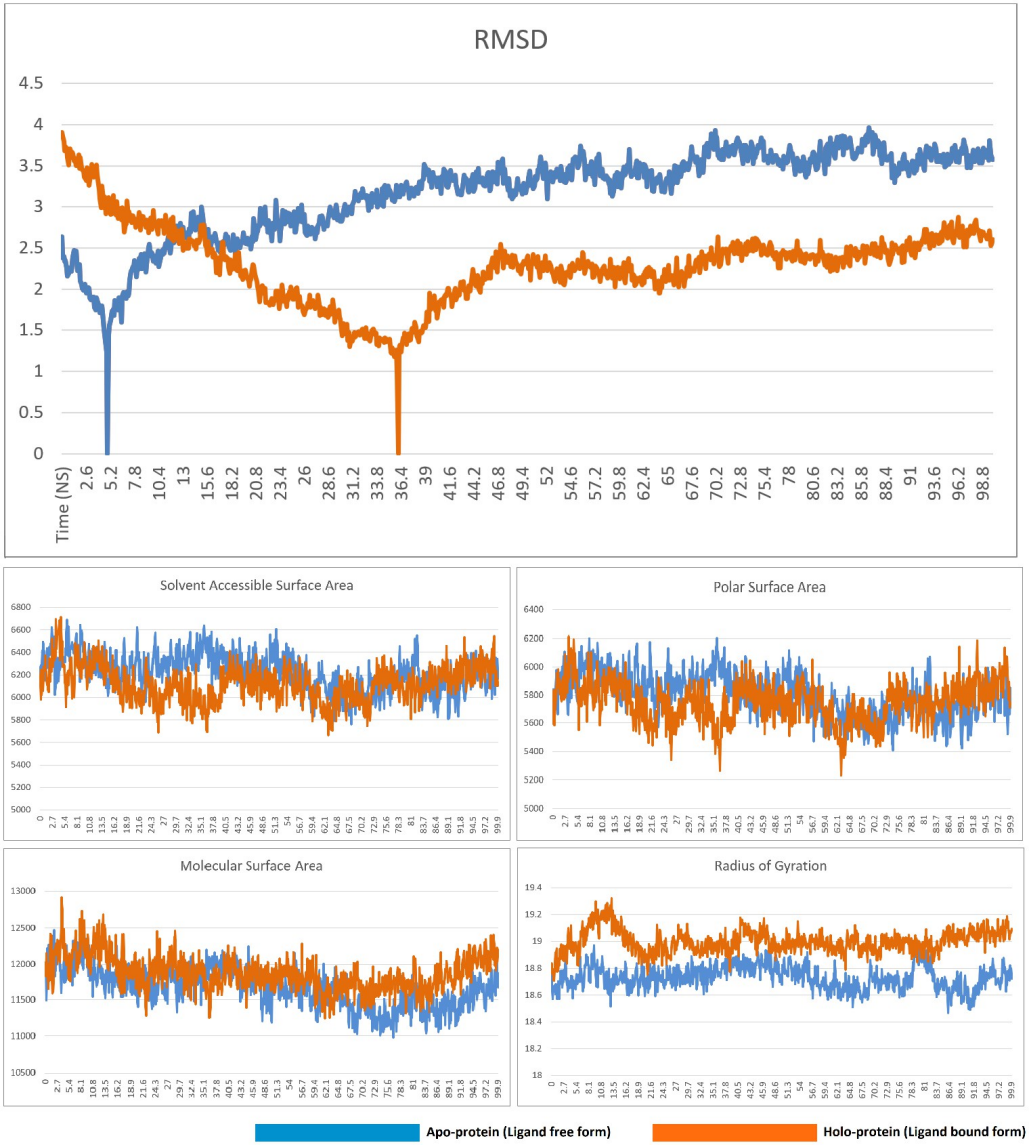
RMSD, solvent accessible surface area, polar surface area, molecular surface area, and radius of gyration of the free and compound 8-bound form of PPAR-α.

Molecular dynamics simulations of the compound 8-PPAR-α complex revealed the complex to be stable. The ligand-free form displayed a sudden downward spike in the RMSD graph near the 5^th^ nanosecond. A similar downward spike was recorded for the complex, albeit later in the trajectory, i.e. near the 36^th^ nanosecond. Following this spike, both RMSD graphs displayed minimal fluctuations, although the RMSD of the ligand-bound form of the protein was generally lower than the ligand-free form. The solvent accessible surface area, polar surface area, and molecular surface area of the apo and the holo form of the protein closely resemble each other, indicating that the protein-ligand complex is as stable as the ligand free form. Ligand-binding results in a radius of gyration graph that is slightly higher that the non-ligand-bound form, but other than this, all other relevant graphs indicate the stability of the complex. The RMSD, solvent accessible surface area, polar surface area, molecular surface area, and radius of gyration of the free and compound 8-bound form of PPAR-α is presented in Fig 22.

High gastrointestinal absorption was predicted for 31 out of the 76 compounds, 13 were predicted to have blood-brain barrier permanent capabilities, and 19 were predicted to be P-glycoprotein substrates. 18 drugs in total were predicted to be CYP-inhibitors of various sorts, of which 6 were predicted to inhibit CYP1A2, 2 CYP2C19, 11 CYP2C9, 5 CYP2D6, and 9 CYP3A4. The ADME data of the phytoconstituents that displayed the 10 highest binding affinity values with either of the two target macromolecules are illustrated in Table 7.

**Table 7:** ADME properties of the phytoconstituents of interest.

Compound ID	GI absorption	BBB permeant	Pgp substrate	CYP inhibition (CYP1A2, CYP2C19, CYP2C9, CYP2D6, CYP3A4)	Lipinski violations	Ghose violations	Veber violations	Egan violations	Muegge violations
C_8	High	No	No	0/5	0	0	0	0	0
C_13	Low	No	Yes	0/5	3	1	1	1	3
C_15	High	No	No	1/5	0	0	0	0	0
C_16	Low	No	Yes	0/5	3	1	1	1	3
C_20	Low	No	Yes	0/5	3	1	1	1	3
C_26	Low	No	No	0/5	0	1	1	1	0
C_27	Low	No	No	0/5	0	1	0	1	0
C_31	Low	No	No	0/5	1	1	1	1	2
C_44	High	No	No	3/5	0	0	0	0	0
C_45	Low	No	No	0/5	2	0	1	1	3
C_48	High	No	No	3/5	0	0	0	0	0
C_50	High	No	No	3/5	0	0	0	0	0
C_51	Low	No	No	2/5	1	0	1	1	2
C_52	High	No	No	3/5	0	0	0	0	0
C_54	High	No	No	3/5	0	0	0	0	0

Of the 76 phytoconstituents, only 20 were predicted not to induce any sort of toxicity. 12 were predicted to be highly carcinogenic, 36 of them were highly immunotoxic, and 2 of them were highly mutagenic. Moderate probability of carcinogenicity, mutagenicity, immunotoxicity, and cytotoxicity were observed in 10, 6, 2, and 1 compound, respectively. 5 compounds belonged to class II (fatal if swallowed), 8 to class III (toxic if swallowed), 13 to class IV (harmful if swallowed), and the rest to classes V and VI. The toxicity data of the phytoconstituents that displayed the 10 highest binding affinity values with either of the two target macromolecules are presented in Table 8.

**Table 8:** Toxicity profile of the phytoconstituents of interest (Toxicity class I: Fatal if swallowed, class II: Fatal if swallowed, class III: Toxic if swallowed, class IV: Harmful if swallowed, class V: May be harmful if swallowed, class VI: Non-toxic).

Compound ID	Predicted toxicity class	Predicted LD50 mg/kg	Toxicity predicted
C_8	3	77	Carcinogenicity**, Immunotoxicity**, Mutagenicity**
C_13	5	5000	Immunotoxicity**
C_15	5	5000	Immunotoxicity**
C_16	5	5000	Immunotoxicity**
C_20	5	5000	Immunotoxicity**
C_26	5	5000	Immunotoxicity**
C_27	5	4000	Immunotoxicity**
C_31	5	3750	-
C_44	5	2500	Cytotoxicity*
C_45	5	5000	-
C_48	5	3919	-
C_50	5	3919	Carcinogenicity*, Mutagenicity*
C_51	3	159	Carcinogenicity*, Mutagenicity*
C_52	5	4000	Carcinogenicity*, Mutagenicity*
C_54	3	159	Carcinogenicity*, Mutagenicity*

Low probability of toxicity*

High probability of toxicity**

## Discussion

In this study, we assessed the hepatoprotective activity of the ethanolic extract of *Gynura procumbens* through standard parameters. In body weight determination, CCl_4_-induced hepatic impairment would limit the body’s capacity to metabolize nutrients, resulting in metabolic imbalance and substantial weight loss. Negative control rats gained terminal weight, demonstrating proper metabolic equilibrium [Fig 1]. Only silymarin-treated groups showed the opposite result, indicating its detrimental effect on healthy rats’ growth rate and body weight. 70% ethanolic extract of *G*. *procumbens* corrected CCl_4_-induced weight loss in a dose-dependent manner, demonstrating the extract’s promise in restoring standard growth rate and body weight in diseased rats. We have found prior studies that *G*. *procumbens* ethanolic extract restored body weight in diseased mice [79, 80]. Increases in body weight after treatment with plant extracts were also found in the case of *Phytolacca dodecandra* [1]. According to a study conducted in Bangladesh, we also found that treatment with *G*. *procumbens* ethanolic extract resulted in consistent recovery of sick rats’ body weights [81]. Both the aqueous and 90% ethanolic extracts of G. procumbens efficiently recovered body weights in sick rats in a dose-dependent manner [82].

To assess liver and kidney functioning tests, some important markers, i.e., SGPT, SGOT, and ALP for liver and creatinine for kidney, were evaluated among groups [Figs 2–5]. In those tests, the marker mentioned above in the disease control group rose dramatically, showing CCl_4_-induced hepatotoxicity that caused liver and kidney enzymes to seep into the circulation and raise serum enzyme levels. *In vivo* delivery of *G*. *procumbens* to the disease control group caused a dose-dependent radical shift of the CCl_4_-induced changes in biomarkers, providing statistically non-significant (p>0.05) results with the standard treatment (silymarin). Our findings regarding the reversal efficacy of the above-mentioned biomarkers of *G*. *procumbens* ethanolic extract were consistent with other studies that found significant serum enzyme (i.e., SGPT and SGOT for liver and creatinine for kidney) restoration efficacy of *G*. *procumbens* using 100% and 70% ethanolic extract, respectively [80, 81, 83]. Similar types of decline in SGPT, and SGOT, and ALP level were observed in some studies using leaf extracts of *Rhododendron arboreum*, *Beta vulgaris*, *Zanthoxylum armatum*, *Saururus chinensis*, *Curcuma longa*, *Limonium sinense*, *Hemidesmus indicus*, *Premna esculenta*, *Macrocybe gigantea*, and *Argyreia speciosa* [2–11]. However, high doses of *Beta vulgaris* is needed to reduce the SGPT level in the same amount as silymarin [5]. Creatinine levels were decreased by some other plant extracts like *Syzygium aromaticum* and *Marrubium vulgare* [17, 29].

As illustrated in Fig 6, serum LDH levels and in Figs 7–10, the levels of serum lipids (i.e., HDL, LDL, triglyceride, and total cholesterol) were assessed. *G*. *procumbens* reversed the CCl_4_-induced alterations in serum LDH and lipid profile in a dose-dependent manner like standard silymarin, which proved the potential antihepatotoxic action of *G*. *procumbens*. Other studies also depicted similar results in the case of the plant’s 70% and 80% ethanolic extract, respectively [79–81, 84]. A similar pattern was observed in some other plants like, *Nigella sativa*, *Beta vulgaris*, *Clerodendrum inerme*, *Solanum xanthocarpum*, *Borago officinalis*, *Aegle marmelos and Mentha piperita*, *Phytolacca dodecandra* [1, 2, 4, 5, 30–33].

The DNA fragmentation profiles of the non-CCl_4_ treated groups were pretty consistent with that of the negative control group, inferring that neither silymarin nor *G*. *procumbens* ingestion would cause any discernible deleterious effects on the DNA fragmentation profiles of the healthy individuals [Fig 11]. Additionally, the plant extracts gradually normalized the disease condition in a dose-dependent manner treating the diseased rats with differing doses (e.g., low, medium, and high) of silymarin or *G*. *procumbens* significantly lowered the elevated levels of serum γ-GT enzyme [Fig 12]. These findings were similar to those of previous studies despite the difference that they performed their experiment employing an aqueous extract of *G*. *procumbens* [85]. The lowering of γ-GT levels was also found in the *Mentha piperita* and *Garcinia kola* plant extracts [30, 34].

In the assessment of oxidative stress, administration of fluctuating doses (low, medium, and high) of *G*. *procumbens* to the disease control group was noticed to cause an effective reversal in CCl_4_-induced alterations in the levels of SOD, CAT, and MDA enzymes that evinced the dose-dependent efficacy of *G*. *procumbens* in attenuating CCl_4_-induced oxidative stress. The same type of results has been found with the following plants- *Macrocybe gigantea*, *Polygonum cuspidatum*, *Marrubium vulgare*, and *Terminalia arjuna* [3, 16, 17, 35].

The findings of the histopathological analysis are closely allied with the data acquired from the assessment of biochemical parameters. The recovered liver histology of *G*. *procumbens* treated diseased rats denoted the remarkable potentiality of the plant extract against CCl4-induced hepatic impairment. High doses of our extract have significantly minimized the effect of swelling of hepatocytes, cholestasis, microvesicular and macrovesicular steatosis, lobular inflammation, portal inflammation, and dilatation of sinusoids, etc. Such outcomes were found in several prior studies conducted on *Casuarina equisetifolia*, *Cajanus cajan*, *Glycosmis pentaphylla*, *Bixa orellana*, *Trichosanthes cucumerina*, *Hygrophila spinosa and Cassia occidentalis* [86–88].

Furthermore, the current study’s findings revealed the superiority of *G*. *procumbens* in minimizing some of the side effects of antihepatotoxic drugs currently available on the market. Many commercially available drugs used to treat various hepatic disorders showed hepatotoxic effects in several cases. Unlike some conventional therapies, orally given *Gynura procumbens* leaf extract was observed to show clinical safety and less toxicity upon long-term use. Side effects like drug-induced liver damage and hepatotoxicity were also lower in *G*. *procumbens-treated* rats [89–100].

In the molecular docking analysis, *G*. *procumbens* phytoconstituents performed poorly against TGF-β1 compared to the control drug galunisertib. Galunisertib scored a binding affinity value of -11.5 Kcal/mole, while the highest-scoring compound, a flavonoid, compound 50, or luteolin scored -10.3. The highest-scoring phenolic compound, compound 20, or 4, 5-dicaffeoylquinic acid, scored -10.1. This difference may be owed to the structural disparities between these molecules and their subsequent abilities to occupy the binding pocket of the macromolecule.

**Fig 23:**
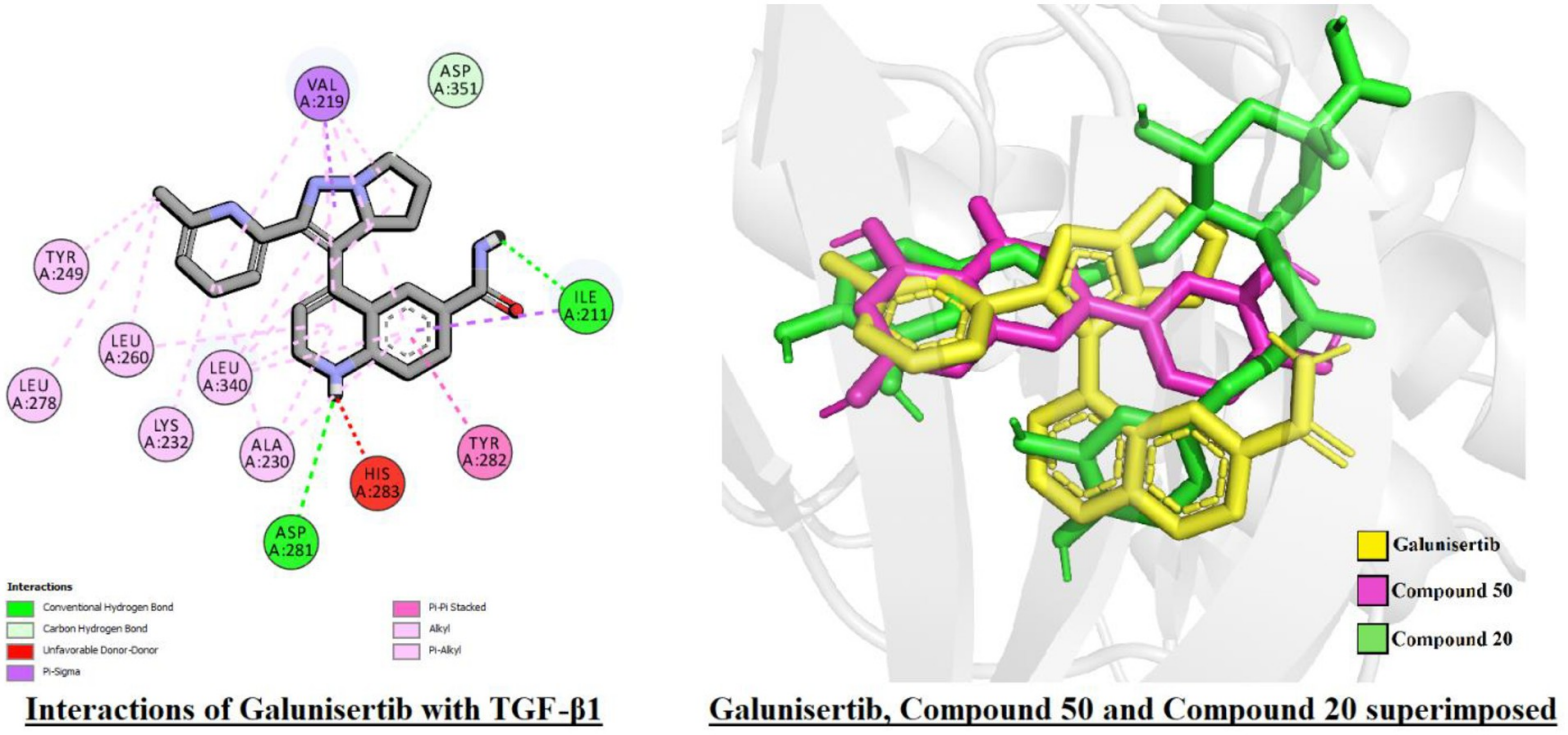
Panel (1) shows interactions of Galunisertib with TGF-β1 receptor. Panel (2) Galunisertib, Compound 50, and Compound 20 superimposed in their binding poses with the macromolecule TGF-β1. Galunisertib has been marked in yellow, Compound 50 in pink, and Compound 20 in green.

Fig 23’s panel 2 shows the steric disparity between the three molecules. Galunisertib has two conjoined pentacyclic ring structures, the second of which has two branches attached to it, one of which contains a single hexacyclic ring and the other two conjoined hexacyclic rings. This unique structure allows galunisertib to form interactions with 12 residues in the binding pocket. While compound 50 has a partially similar steric configuration, it lacks the cyclic side branch and therefore cannot occupy the binding pocket to a similar degree as galunisertib. Compound 20’s steric configuration is more akin to that of galunisertib. However, it lacks cyclic structures in a number of positions compared to galunisertib. Moreover, a cyclic extension at the junction point contributes little to ligand-macromolecule interactions but likely cause steric hindrance, resulting in a lower binding affinity than the control. Steric similarities are of utmost importance in computational drug design, and increasing the steric similarities through structural modification may result in better binding affinities with TGF-β1 [101–104].

Molecular docking analysis revealed that the phytoconstituents performed considerably better than the control bezafibrate against macromolecule PPAR-α, with 26 phytoconstituents scoring better than the control (B.A.: -7.6 Kcal/mole). A few high-scoring compounds (compounds 25, 26, and 38) did not share any interaction residues with the control, but this was not observed across the library. Flavonoids and phenolic compounds comprised most of the top-scoring ligands for both macromolecules. In the case of TGF-β1, all 10 of the best-scoring phytoconstituents belonged to either of these classes. In the case of PPAR-α, 9 of the top 10 best-scoring phytoconstituents were flavonoids or phenolic compounds. Flavonoids and phenolic compounds have long been known to exert hepatoprotective activity, which was also reflected here [105–107].

Gynura Procumbens has diverse pharmacological effects due to its vast phytochemical constituents; which includes alkaloids, tannins, saponins, essential oils, steroids, triterpenoids, proteins, flavonoids, and polysaccharides, etc. These elements have considerable antioxidant properties. However, predominant flavonoids kaempferol-3-O-rutinoside, and myricetin have great scavenging capacity of DPPH and ABTS+ radicals. Since CCl4 increases cellular necrosis of the liver by generating free radicals, it may be postulated that the presence of flavonoids in *Gynura procumbens* scavenges these free radicals and gradually reduces the cellular necrosis of the liver [108–111]. Overall, *Gynura procumbens* might be a promising, clinically applicable, natural counteraction against drug-induced side effects when long-term or multiple drug therapy is required for a potent hepatoprotective action.

## Conclusion

The current study on *G*. *procumbens* in rat models provides new insight into hepatoprotective activity. A decrease in SGPT, SGOT, ALP, creatinine, TC, LDL, triglycerides (TG), SOD, MDA, DNA fragmentation ranges, γ-GT levels, and an increase in HDL level in CCl_4-_treated rats by both standard drug silymarin and *G*. *procumbens* leaf extract demonstrated a potential hepatoprotective activity of the plant extract. From the histopathological study, it is also shown that *G*. *procumbens* can reverse the pathological state after treating with CCl_4_. In molecular docking analysis, several bioactive molecules showed optimistic binding affinity to receptor proteins, and the ADMET study displayed their drug-likeness characters. The results of both *in vivo* and *in silico* studies give a ray of hope to consider *G*. *procumbens* leaf extract as a possible wellspring of the hepatoprotective drug to be used as an alternative medicine for treating hepatotoxicity. However, further studies are needed to establish the responsible active compounds and their possible mode of action.
